# Calponin 2 regulates ketogenesis to mitigate acute kidney injury

**DOI:** 10.1172/jci.insight.170521

**Published:** 2023-11-08

**Authors:** Yuan Gui, Zachary Palanza, Priya Gupta, Hanwen Li, Yuchen Pan, Yuanyuan Wang, Geneva Hargis, Donald L. Kreutzer, Yanlin Wang, Sheldon I. Bastacky, Yansheng Liu, Silvia Liu, Dong Zhou

**Affiliations:** 1 Division of Nephrology, Department of Medicine, University of Connecticut School of Medicine, Farmington, Connecticut, USA.; 2 Departments Statistics, Kenneth P. Dietrich School of Arts and Sciences, University of Pittsburgh, Pittsburgh, Pennsylvania, USA.; 3 Department of Bioinformatics and Computational Biology, University of Texas MD Anderson Cancer Center, Houston, Texas, USA.; 4 University of Connecticut School of Medicine, Farmington, Connecticut, USA.; 5 Department of Surgery, University of Connecticut School of Medicine, Farmington, Connecticut, USA.; 6 Department of Pathology, University of Pittsburgh School of Medicine, Pittsburgh, Pennsylvania, USA.; 7 Yale Cancer Biology Institute and; 8 Department of Pharmacology, School of Medicine, Yale University, New Haven, Connecticut, USA.

**Keywords:** Nephrology, Apoptosis, Mouse models, Pericytes

## Abstract

Calponin 2 (CNN2) is a prominent actin stabilizer. It regulates fatty acid oxidation (FAO) by interacting with estrogen receptor 2 (ESR2) to determine kidney fibrosis. However, whether CNN2 is actively involved in acute kidney injury (AKI) remains unclear. Here, we report that CNN2 was induced in human and animal kidneys after AKI. Knockdown of CNN2 preserved kidney function, mitigated tubular cell death and inflammation, and promoted cell proliferation. Distinct from kidney fibrosis, proteomics showed that the key elements in the FAO pathway had few changes during AKI, but we identified that 3-hydroxymethylglutaryl-CoA synthase 2 (Hmgcs2), a rate-limiting enzyme of endogenous ketogenesis that promotes cell self-renewal, was markedly increased in CNN2-knockdown kidneys. The production of ketone body β-hydroxybutyrate and ATP was increased in CNN2-knockdown mice. Mechanistically, CNN2 interacted with ESR2 to negatively regulate the activities of mitochondrial sirtuin 5. Activated sirtuin 5 subsequently desuccinylated Hmgcs2 to produce energy for mitigating AKI. Understanding CNN2-mediated discrete fine-tuning of protein posttranslational modification is critical to optimize organ performance after AKI.

## Graphical abstract

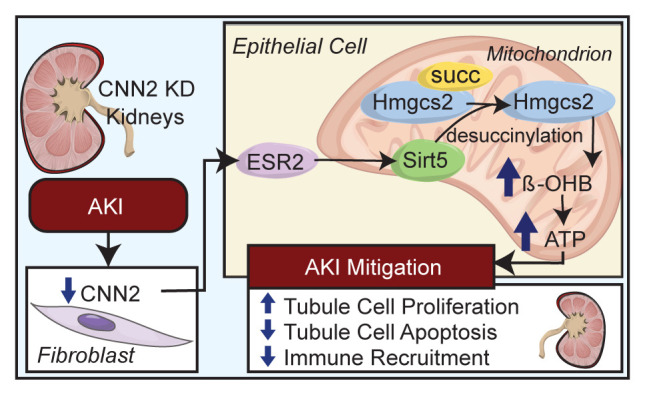

## Introduction

Calponin (CNN) is a calcium-binding protein. It has 3 isoforms encoded by homologous genes in vertebrates, *CNN1*, *CNN2*, and *CNN3* (1). These isoforms are conserved proteins, whereas CNN2 has diverged from CNN1 and CNN3 in the C-terminal variable region. First identified in chicken gizzard smooth muscle (2), CNN2 is responsible for binding actin-binding proteins and tonically inhibiting the myosin ATPase activity in smooth muscle. Over the past several years, sporadic reports revealed the translational value of CNN2 from the perspective of cell mechanics, such as inhibiting tumor metastasis (3). Emerging evidence indicates that CNN2 is linked to various fibrotic processes, such as postoperative peritoneal adhesions and kidney fibrosis (4, 5). In chronic kidney disease (CKD), CNN2 regulates energy metabolism by interacting with estrogen receptor 2 (ESR2) to control fibrosis progression (5). Therefore, it would be worthwhile to fully dissect the role of CNN2 in acute kidney injury (AKI), given that AKI and CKD are closely intertwined but have distinct pathogenesis.

AKI is a refractory clinical syndrome that refers to sudden kidney function loss within a few hours or days. Thus far, there are no effective strategies to predict, prevent, or mitigate AKI, though the field has a consensus that kidney tubules are the epicenter of damage after AKI. Tubule repair requires high energy that depends on metabolic processes to generate ATP. Consequently, the extent of tubule repair is highly associated with AKI prognosis. During tubule repair, neighboring fibroblasts emerge as orchestrators of a favorable kidney local microenvironment (KLM) (6). In CKD, CNN2 is predominantly expressed in fibroblasts/pericytes, and knockdown of CNN2 alleviates kidney fibrosis by enhancing fatty acid oxidation (FAO) (5). Surprisingly, after AKI, our proteomics showed that CNN2 knockdown had a minor effect on FAO but markedly induced 3-hydroxy-3-methylglutaryl-CoA synthase 2 (Hmgcs2) in the kidney, a rate-limiting enzyme in ketogenesis. Accordingly, we proposed determining the importance of ketone bodies as a fuel source in AKI mitigation and how CNN2 regulates its production.

Ketone bodies are 3 small water-soluble lipid molecules (acetoacetate, acetone, and β-hydroxybutyrate [β-OHB]) mainly produced in the liver by breaking down fatty acids (7). They deliver energy from the liver to peripheral tissues during fasting or prolonged exercise (8). In the steps of ketogenesis, Hmgcs2 catalyzes acetyl-CoA and acetoacetyl-CoA into HMG-CoA, and then HMG-CoA lyase liberates acetoacetate. Acetoacetate is the common precursor of the other circulating ketone bodies, acetone and β-OHB (9). Most acetoacetate is further metabolized to β-OHB. β-OHB is the most abundant circulating ketone body and can reduce oxidative stress, suppress inflammation, and protect against cell death or senescence (7, 10–12). As a mitochondrial enzyme controlling β-OHB production, Hmgcs2 is often regulated by transcription or posttranslational modification (PTM). For instance, sirtuins can deacetylate or desuccinylate Hmgcs2 during fasting or tumorigenesis (13–15). With regard to the above, we hypothesized that CNN2 regulates Hmgcs2-mediated ketogenesis to create an appropriate microenvironment for preventing or mitigating AKI. Deciphering the mechanism is critical for supplementing a previously unrecognized piece of the puzzle to AKI pathogenesis and designing efficient therapeutic strategies.

## Results

### CNN2 is expressed in mouse and human kidneys after AKI.

**Figure 1:**
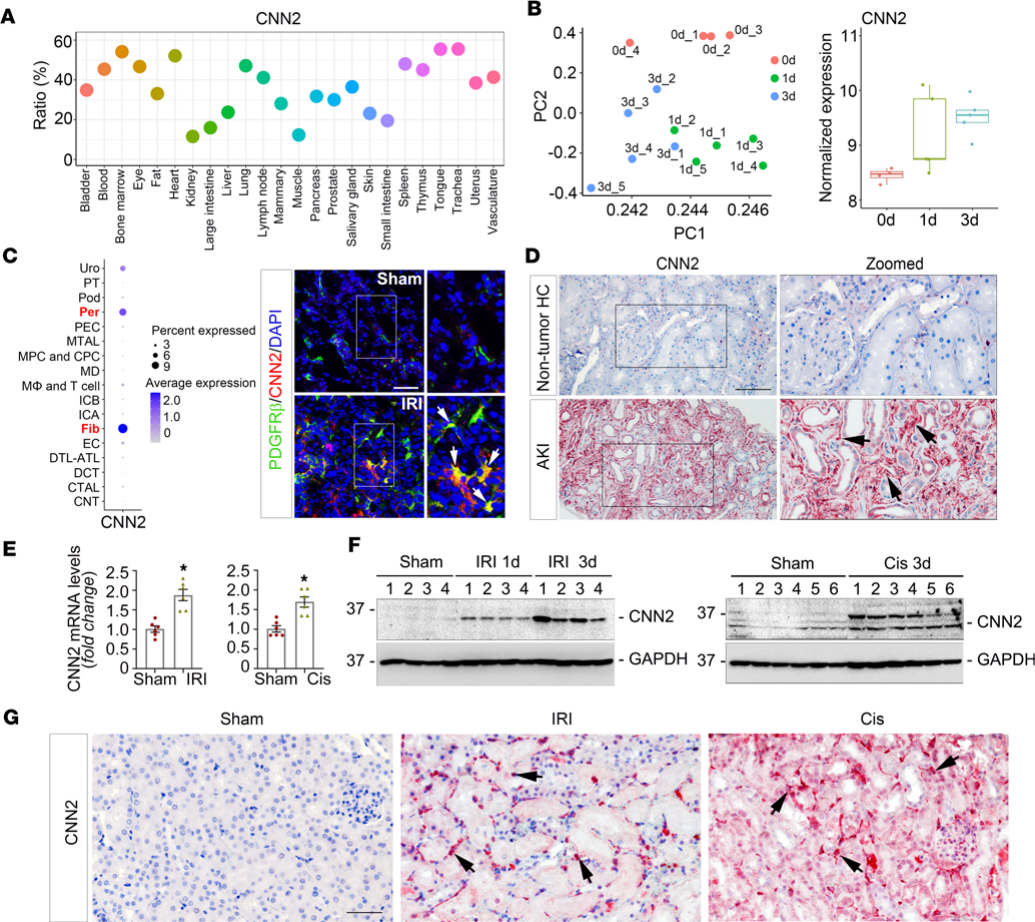
CNN2 inductions in the AKI kidneys. (**A**) The percentage of cells expressing CNN2 in multiple organs based on a single-cell transcriptomic atlas of humans. (**B**) Bulk RNA sequencing revealed CNN2 was induced in the mouse kidneys after renal IRI at 1 and 3 days. Left panel: principal component analysis (PCA) colored by different time points. Right panel: normalized CNN2 expression at 0, 1, and 3 days. (**C**) Single-nucleus RNA sequencing showed CNN2 is predominantly expressed by fibroblasts and pericytes after IRI. Costaining for CNN2 (red) and the marker for fibroblasts/pericytes, PDGFR-β (green). Scale bar, 25 μm. Arrows indicate positive staining. (**D**) Representative immunohistochemical staining images showed CNN2 expression in nontumor normal human kidney and kidney biopsy specimens from patients with AKI. Boxed areas are zoomed (original magnification, 20×). Arrows indicate positive staining. Scale bar, 50 μm. (**E**) Quantitative real-time PCR analysis revealed the levels of *CNN2* mRNA in the diseased kidneys after IRI and cisplatin injection, respectively. *P < 0.05 (*n* = 6). (**F**) Western blot assays show CNN2 protein expression in the diseased kidneys after IRI (left panel) and cisplatin (right panel), respectively. Numbers indicate individual animals within each group. (**G**) Immunohistochemical staining showed the distribution of CNN2 in mouse kidneys after IRI and cisplatin. Original magnification, 40×. Graphs are presented as means ± SEM. Differences between groups were analyzed using unpaired *t* tests or ANOVA followed by the Student-Newman-Keuls test. IRI, ischemia/reperfusion injury; PDGFR-β, PDGF receptor-β.

First, to understand the distribution of CNN2 in different organs, we performed deep analyses by mining a public human single-cell transcriptomic database (16). As illustrated in Figure 1A, CNN2 is widely expressed in multiple organs. For example, more than half of the cells in the bone marrow, heart, tongue, and trachea show high expression levels of CNN2. Among all the cells in a healthy kidney, around 11.6% of the cells express CNN2, which is comparatively lower than the other organs. However, our bulk RNA-sequencing study revealed that CNN2 was markedly induced in the mouse-diseased kidneys at day 1 and 3 after renal ischemia/reperfusion injury (IRI) (Figure 1B). A separate analysis using single-nucleus RNA sequencing showed that CNN2 was enriched in fibroblasts and pericytes after IRI (Figure 1C) (17). To verify the cellular specificity of CNN2, we costained CNN2 (shown in red) with fibroblast/pericyte marker PDGF receptor-β (PDGFR-β; green), macrophage marker F4/80 (green), and endothelial marker CD31 (green) in the mouse kidneys after IRI. CNN2 was mainly expressed by PDGFR-β^+^ fibroblasts/pericytes after AKI (Figure 1C, right panel). Little inductions were seen in F4/80^+^ macrophages, and there was no overlap with CD31^+^ endothelium in the ischemic kidneys (Supplemental Figure 1; supplemental material available online with this article; https://doi.org/10.1172/jci.insight.170521DS1). To establish the clinical relevance of CNN2 expression and human AKI, we performed immunohistochemical staining for CNN2 in kidney biopsy specimens from patients with AKI. Compared with the expression in the nontumor normal human kidneys, CNN2 was upregulated in the kidney interstitium in patients with AKI (Figure 1D). Then, we examined CNN2 expression in the kidneys obtained from 2 well-characterized AKI mouse models induced by IRI and cisplatin administration, respectively. After IRI or cisplatin injection, CNN2 mRNA levels were increased in the diseased kidneys compared with sham mice (Figure 1E). Additionally, Western blot assay demonstrated that CNN2 protein was elevated in AKI kidneys (Figure 1F), and immunohistochemical staining showed that CNN2 was consistently induced in the kidney interstitium (Figure 1G).

### Knockdown of CNN2 mitigates AKI in mice.

**Figure 2:**
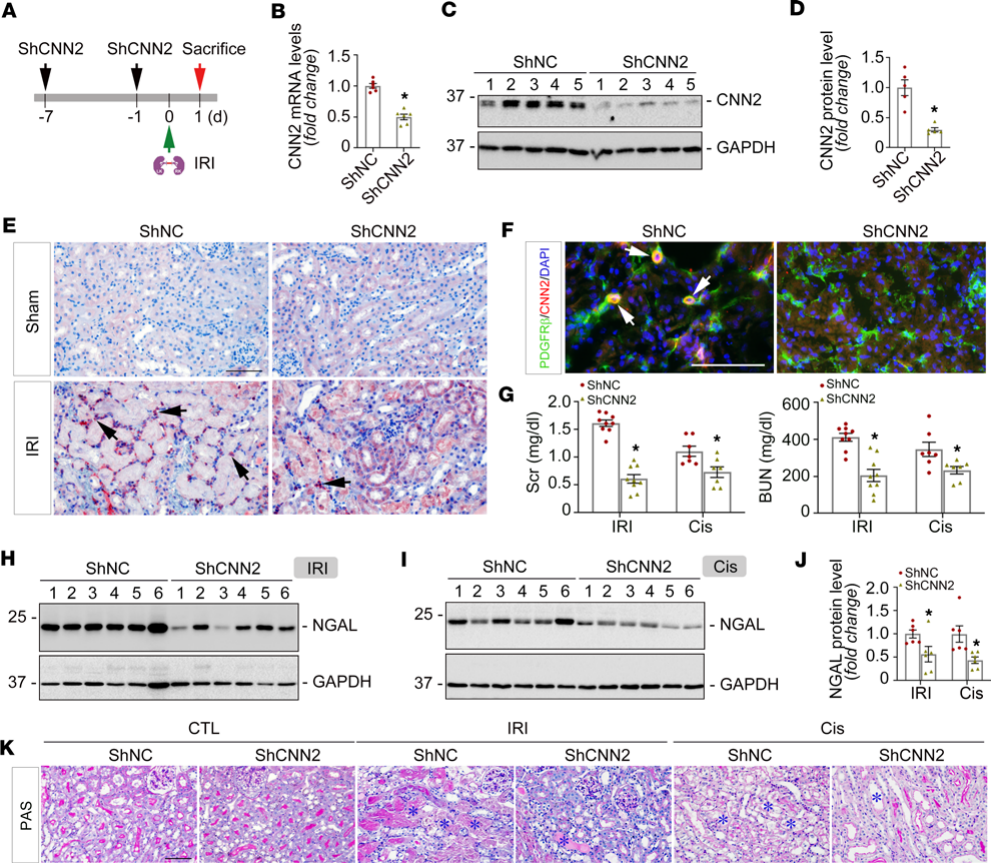
Knockdown of CNN2 mitigates ischemic AKI. (**A**) Experiment design. ShCNN2 plasmid was administrated in mice 7 days (d) and 1 d before IRI, respectively. The mice were sacrificed at 1 d after IRI. (**B**) Quantitative real-time PCR analysis showed the changes of *CNN2* mRNA levels in kidneys of ShNC and ShCNN2 mice after IRI. *P < 0.05 (*n* = 6). (**C** and **D**) Western blot assay demonstrated CNN2 protein expression in kidneys of ShNC and ShCNN2 mice after IRI (**C**), and quantified data were presented (**D**). Numbers indicate individual animals within each group. *P < 0.05 (*n* = 5). (**E**) Immunohistochemical staining showed CNN2 expression and distribution in kidneys of ShNC and ShCNN2 mice after IRI. Scale bar, 25 μm. Arrows indicate positive staining. (**F**) Costaining for CNN2 (red) and PDGFR-β (green) in the kidneys demonstrated CNN2 induction was largely abolished in fibroblasts/pericytes. Scale bar, 50 μm. Arrows indicate positive staining. (**G**) Serum creatinine (Scr) and blood urea nitrogen (BUN) levels in ShNC and ShCNN2 mice at 1 d after IRI or 3 d after cisplatin injection. *P < 0.05 (*n* = 7–9). (**H**–**J**) Representative Western blots (**H** and **I**) and quantified data (**J**) of NGAL protein expression in ShNC and ShCNN2 kidneys at 1 day after IRI or 3 days after cisplatin injection. Numbers indicate individual animals within each group. *P < 0.05 (*n* = 6). (**K**) The changes of kidney histology as shown by periodic acid–Schiff (PAS) staining in ShNC and ShCNN2 mice at 1 d after IRI or 3 d after cisplatin injection. Scale bar, 50 μm. Blue asterisks indicate injured tubules. Graphs are presented as means ± SEM. Differences between groups were analyzed using unpaired *t* tests.

To explore the role of CNN2 in vivo, we knocked down *CNN2* in IRI- and cisplatin-induced AKI models, respectively. As depicted in Figure 2A and Supplemental Figure 2A, ShCNN2 plasmid was administered to mice at 2 time points before IRI surgery or cisplatin injection: once 7 days before and again 1 day before. The mice were then sacrificed 1 day after IRI or 3 days after cisplatin injection. Compared with the vehicle group, qPCR and Western blot assays verified that CNN2 was remarkably decreased in ShCNN2 kidneys after AKI (Figure 2, B–D, and Supplemental Figure 2, B and C). Immuno- and co-immunostaining further showed decreased CNN2 in PDGFR-β^+^ fibroblasts/pericytes of the AKI kidneys (Figure 2, E and F, and Supplemental Figure 2D).

Then, we examined whether knockdown of CNN2 affects kidney function after AKI. As shown in Figure 2G, compared with vehicle animals (ShNC), serum creatinine (Scr) and blood urea nitrogen (BUN) levels were reduced in ShCNN2 mice after AKI in both models. Western blot assay demonstrated that neutrophil gelatinase-associated lipocalin (NGAL), a classic tubular injury marker (18), was decreased in ShCNN2 kidneys (Figure 2, H–J). Consistently, periodic acid–Schiff (PAS) staining showed that morphological changes in ShCNN2 kidneys had less brush border loss and reduced intratubular proteinaceous casts (Figure 2K and Supplemental Figure 2E). To further determine whether knockdown of CNN2 affects kidney outcomes at the repair phase, we constructed an additional moderate ischemic AKI model with lower mortality (Supplemental Figure 3A). At 7 days after the moderate IRI, compared with vehicles, CNN2-knockdown mice exhibited preserved Scr and BUN levels, improved histological changes, reduced NGAL, and increased proliferating cell nuclear antigen (PCNA) protein expression in the ischemic kidneys (Supplemental Figure 3, B–K). Thus, our results suggested that knockdown of CNN2 mitigated AKI and could eventually improve kidney outcomes.

### Knockdown of CNN2 reduces tubular cell death and promotes cell proliferation.

**Figure 3:**
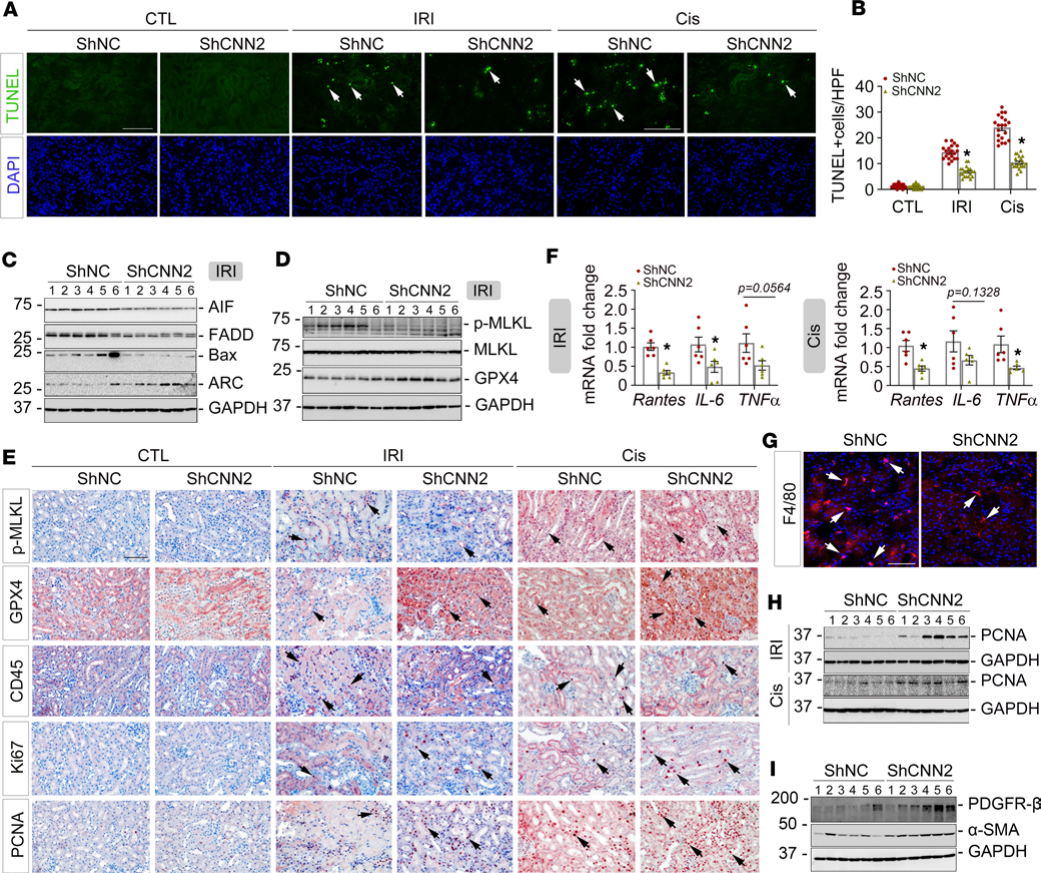
Knockdown of CNN2 attenuates tubular cell death and kidney inflammation in AKI. (**A** and **B**) Representative micrographs (**A**) and quantitative data (**B**) of TUNEL staining in ShNC and ShCNN2 mouse kidneys 1 day after IRI or 3 days after cisplatin injection. Arrows indicate apoptotic cells. DAPI is a nuclear counterstain. Scale bar, 50 µm. *P < 0.05 (*n* = 5). (**C**) Western blot assay demonstrated the expression of AIF, FADD, Bax, and ARC in ShNC and ShCNN2 mouse kidneys at 1 day after IRI (*n* = 6). (**D**) Western blot assay demonstrated p-MLKL, MLKL, and GPX4 expression in ShNC and ShCNN2 mouse kidneys at 1 day after IRI. (**E**) Representative immunohistochemical staining micrographs for p-MLKL, GPX4, CD45, Ki67, and PCNA in ShNC and ShCNN2 mouse kidneys 1 day after IRI or 3 days after cisplatin injection. Scale bar, 50 µm. Arrows indicate positive staining. (**F**) Quantitative real-time PCR analyses revealed the mRNA abundance of Rantes, IL-6, and TNF-α in ShNC and ShCNN2 mouse kidneys 1 day after IRI or 3 days after cisplatin injection. *P < 0.05 (*n* = 6). (**G**) Immunofluorescence staining showed F4/80^+^ macrophages in ShNC and ShCNN2 mouse kidneys 1 day after IRI. Scale bar, 50 µm. Arrows indicate positive staining. (**H**) Western blot assays demonstrated PCNA expression in ShNC and ShCNN2 mouse kidneys at 1 day after IRI or 3 days after cisplatin injection. (**I**) Western blot assays demonstrated PDGFR-β and α-SMA expression in the kidneys from ShNC and ShCNN2 mice 1 day after IRI. For all Western blot panels, numbers indicate individual animals within each group. Graphs are presented as means ± SEM. Differences between groups were analyzed using unpaired *t* tests or ANOVA followed by the Student-Newman-Keuls test. AIF, apoptosis inducing factor; ARC, apoptosis repressor with caspase recruitment domain; FADD, Fas-associated protein with death domain; p-MLKL, phosphorylated mixed lineage kinase domain-like protein; GPX4, glutathione peroxidase 4; α-SMA, α–smooth muscle actin.

Because tubules are the epicenter of damage after AKI, we then examined if CNN2 knockdown affects tubular cell death in the injured kidneys, such as apoptosis, necrosis, or ferroptosis. First, employing terminal deoxynucleotidyl transferase–mediated dUTP nick end labeling (TUNEL) staining, we identified the number of TUNEL-positive tubular cells was decreased in ShCNN2 mice compared with vehicle in the 2 AKI models (Figure 3, A and B). Western blot assays demonstrated that pro-apoptosis proteins –– including apoptosis inducing factor (AIF), Fas-associated protein with death domain (FADD), and Bax –– were reduced, while apoptosis repressor with caspase recruitment domain (ARC) was increased in ShCNN2 kidneys (Figure 3C, Supplemental Figure 4A, and Supplemental Figure 5, A and B). Meanwhile, reduced phosphorylated-mixed lineage kinase domain-like protein (p-MLKL) and increased glutathione peroxidase 4 (GPX4), the proteins that reflect the status of cell necroptosis and ferroptosis, respectively, were detected in ShCNN2 kidneys by Western blot assay and immunohistochemical staining after ischemic AKI (Figure 3, D and E, and Supplemental Figure 4B). Interestingly, there were minor changes of p-MLKL in ShCNN2 kidneys after cisplatin-induced AKI (Figure 3E and Supplemental Figure 5, A and B).

We also assessed the inflammatory response in the diseased kidneys of 2 AKI models. Compared with vehicles, qPCR analysis revealed decreased mRNA abundances of secreted cyto-chemokines, including *Rantes*, *IL-6*, and *TNF-*α, in ShCNN2 kidneys after AKI (Figure 3F). Immunostaining consistently indicated fewer infiltrated CD45^+^ monocytes (Figure 3E, Supplemental Figure 4C, and Supplemental Figure 5C) and F4/80^+^ macrophages (Figure 3G and Supplemental Figure 4D) in ShCNN2 kidneys. Upon AKI, surviving tubular cells undergo proliferation to facilitate kidney repair. Western blot assay demonstrated that, compared with vehicle kidneys, PCNA was upregulated in ShCNN2 kidneys after AKI (Figure 3H and Supplemental Figure 6A). Immunohistochemical staining further showed that PCNA- and Ki67-positive tubular cells were increased in ShCNN2 kidneys (Figure 3E and Supplemental Figure 6B) from our 2 AKI models. Interestingly, knockdown of CNN2 also induced PDGFR-β^+^ or α–smooth muscle actin–positive (α-SMA^+^) fibroblasts/pericytes’ early activation in the kidney after AKI (Figure 3I and Supplemental Figure 4E). Fibroblasts’ early and transient activation has been demonstrated to play a protective role in mitigating AKI (6). Of note, the majority of these activated fibroblasts were kidney-resident cells because we did not see marked changes in other fibroblast-originated resources, such as the epithelial-mesenchymal transition, between vehicle and ShCNN2 mice (Supplemental Figure 4, F–H).

### Proteomics identifies Hmgcs2-mediated ketogenesis as key to mitigate AKI after CNN2 knockdown.

**Figure 4:**
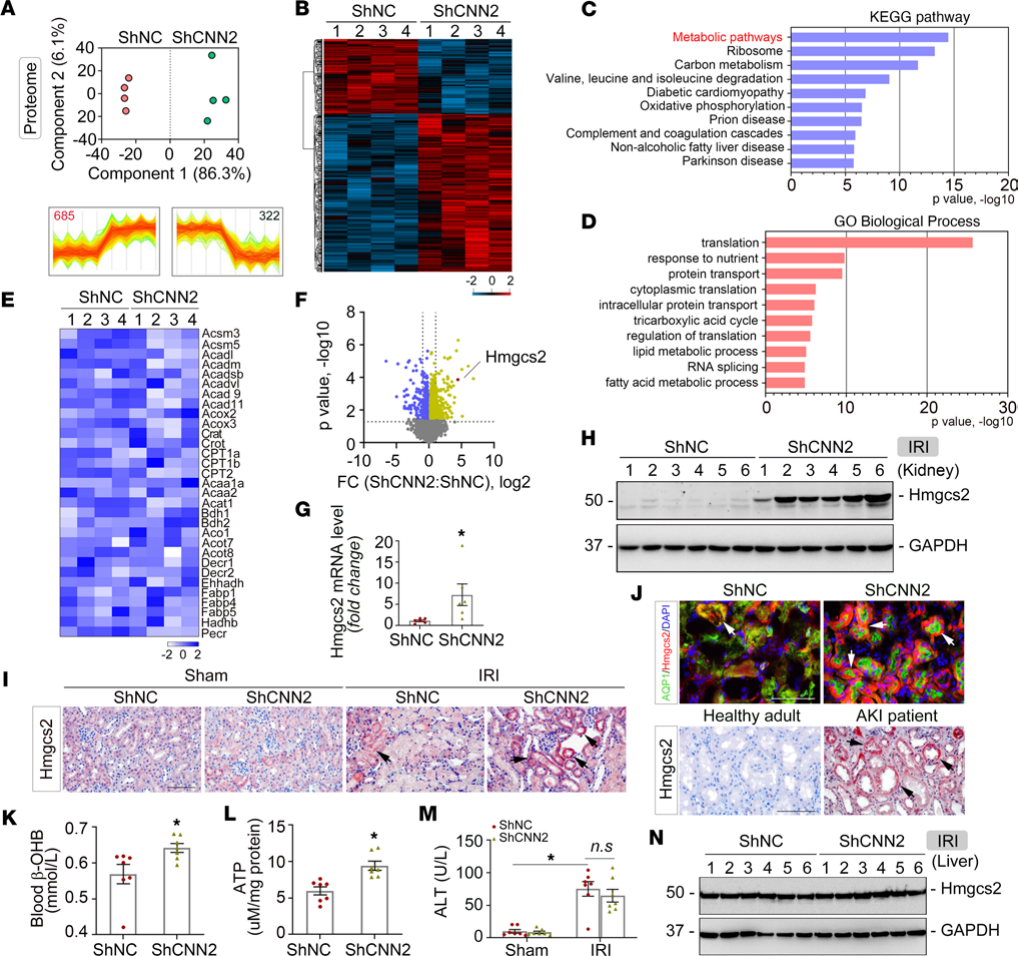
Global proteomics reveals CNN2 knockdown increases Hmgcs2-mediated ketogenesis after AKI. After IRI at 1 day, (**A**) principal component analysis of global proteomes from ShNC and ShCNN2 kidneys. (**B**) Heatmap of *t* test significant proteins. Two clusters of proteins with different patterns of abundance profiles are highlighted. (**C**) Kyoto Encyclopedia of Genes and Genomes (KEGG) pathway enrichment analysis highlighted upregulated pathways in ShNC and ShCNN2 mouse kidneys. (**D**) Gene Ontology (GO) biological process terms in each cluster of proteins are plotted with their names and significance. (**E**) Heatmap of the key components in the fatty acid oxidation pathway in ShNC and ShCNN2 mouse kidneys. (**F**) Volcano plot showed the differential proteins between ShNC and ShCNN2 kidneys. Up- and downregulated proteins (fold-change, FC) are colored in yellow and blue, respectively. (**G**) Quantitative real-time PCR revealed *Hmgcs2* mRNA levels in ShNC and ShCNN2 mouse kidneys. *P < 0.05 (*n* = 6). (**H**) Western blot assay demonstrated Hmgcs2 levels in ShNC and ShCNN2 mouse kidneys. (**I** and **J**) Immunohistochemical staining (**I**) showed Hmgcs2 expression in ShNC and ShCNN2 mouse kidneys. Costaining for Hmgcs2 (red) and aquaporin 1 (AQP1, green) in the kidneys (**J**, upper panel). Immunohistochemical staining showed Hmgcs2 expression in the kidney biopsy specimens from patients with AKI (**J**, lower panel). Scale bar, 50 µm. Arrows indicate positive staining. (**K**–**M**) ELISA showed the levels of β-OHB in blood (**K**), ATP in total kidney tissue (**L**), and alanine transaminase (ALT) in blood (**M**) from ShNC and ShCNN2 mice. *P < 0.05 (*n* = 7). (**N**) Western blot assay demonstrated the expression of Hmgcs2 protein in the liver from ShNC and ShCNN2 mice. For Western blot panels, numbers indicate individual animals within each group. Graphs are presented as means ± SEM. Differences between groups were analyzed using unpaired *t* tests or ANOVA followed by the Student-Newman-Keuls test.

To better understand the molecular mechanisms of how CNN2 knockdown mitigates AKI, we employed a label-free quantitative approach to profile the proteome landscape of ShNC and ShCNN2 kidneys. PCA clearly classified ShNC and ShCNN2 kidneys according to their genotype (Figure 4A). A 2-tailed *t* test identified 1,007 differentially expressed proteins (permutation FDR 0.05) between ShNC and ShCNN2 mice after IRI. Compared with ShNC kidneys, 685 and 322 proteins were up- and downregulated in ShCNN2 kidneys (Figure 4B). The proteomic measurements were generally reproducible as the protein intensity distribution was similar, and the Pearson correlation of the 4 biological replicates of each group was 0.95 or higher (Supplemental Figure 7, A and B). Intriguingly, Kyoto Encyclopedia of Genes and Genomes (KEGG) enrichment and Gene Ontology (GO) analyses indicated that metabolic pathways were the most affected biological processes in shCNN2 diseased kidneys (Figure 4, C and D). Our earlier study showed that CNN2 knockdown activated the FAO pathway in CKD (5). Surprisingly, after AKI, none of the key FAO members were substantially changed between ShNC and ShCNN2 kidneys (Figure 4E). Instead, the volcano plot showed that Hmgcs2, a key rate-limiting enzyme of ketogenesis, was one of the topmost increased proteins in ShCNN2 kidneys compared with vehicle (Figure 4F and Supplemental Figure 7C). This finding indicates that Hmgcs2-mediated ketogenesis may play a key role in AKI mitigation after CNN2 knockdown.

Then, we validated Hmgcs2 expression in the diseased kidneys. qPCR analyses revealed that *Hmgcs2* mRNA was upregulated in ShCNN2 kidneys compared with vehicle (Figure 4G). Western blot assays verified the upregulation of Hmgcs2 in ShCNN2 kidneys (Figure 4H and Supplemental Figure 7D). Consistently, immunostaining showed increased Hmgcs2 in proximal tubules of ShCNN2 kidneys (Figure 4, I and J – upper panel). In human kidney biopsy specimens, Hmgcs2 was also upregulated in the diseased tubules after acute tubular necrosis-induced AKI (Figure 4J, lower panel). We next measured the levels of β-OHB in ShNC and ShCNN2 mice after AKI, given that Hmgcs2 regulates ketogenesis. As shown in Figure 4K, blood β-OHB levels were upregulated in ShCNN2 mice compared with ShNC. Because ketone bodies are the key contributors to intracellular ATP levels in tubular cells, we further detected ATP consumption in the diseased kidneys. ELISA showed that the ATP levels were much higher in ShCNN2 kidneys compared with ShNC (Figure 4L). In the cisplatin-induced AKI model, knockdown of CNN2 also upregulated Hmgcs2 expression in the diseased kidneys (Supplemental Figure 7, E–H). Since the liver is the major organ that produces ketone bodies, we additionally measured the levels of alanine transaminase in blood and Hmgcs2 in the liver. However, there were no changes detected after AKI (Figure 4, M and N), though the liver CNN2 had been knocked down (Supplemental Figure 7I).

**Figure 5:**
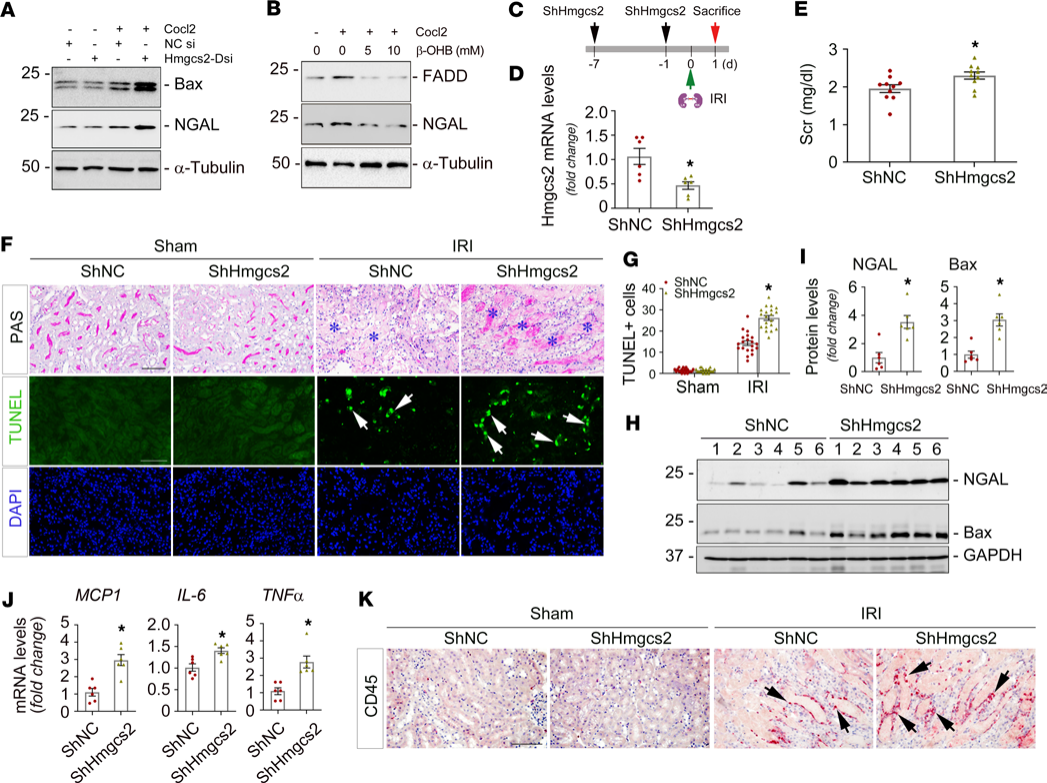
Knockdown of Hmgcs2 aggravates ischemic AKI. (**A**) Normal rat kidney proximal tubular cells (NRK-52E) were transfected with Dicer-substrate siHmgcs2, followed by CoCl2 (400 μM) administration. Western blot assay demonstrated that knockdown of Hmgcs2 increased Bax and NGAL in NRK-52E cells, compared with scramble controls. (**B**) NRK-52E cells were treated with β-OHB at different dosages, followed by CoCl2 (400 µM) administration. Western blot assay showed β-OHB reduced FADD and NGAL induction. (**C**) Experiment design. (**D**) Quantitative real-time PCR (qPCR) analysis showed *Hmgcs2* mRNA levels in ShNC and ShHmgcs2 mouse kidneys after IRI. *P < 0.05 (*n* = 6). (**E**) Serum creatinine (Scr) levels in ShNC and ShHmgcs2 mice after IRI. *P < 0.05 (*n* = 10). (**F**) Periodic acid–Schiff (PAS) staining showed morphological changes in ShNC and ShHmgcs2 mice after IRI. Blue asterisks indicate injured tubules. Representative micrographs of TUNEL staining in the kidneys from ShNC and ShHmgcs2 mice after IRI. Arrows indicate apoptotic cells. DAPI is a nuclear counterstain. Scale bar, 50 µm. (**G**) The quantitative data of apoptotic cells. *P < 0.05 (*n* = 5). (**H** and **I**) Representative Western blots (**H**) and quantified data (**I**) of NGAL and Bax expression in ShNC and ShHmgcs2 mouse kidneys after IRI. Numbers indicate individual animals within each group. *P < 0.05 (*n* = 6). (**J**) qPCR analyses revealed the mRNA abundance of MCP1, IL-6, and TNF-α in the kidneys from ShNC and ShHmgcs2 mice at 1 day after IRI. *P < 0.05 (*n* = 6). (**K**) Representative immunohistochemical staining micrographs for CD45 in ShNC and ShHmgcs2 mouse kidneys after IRI. Scale bar, 50 µm. Arrows indicate positive staining. Graphs are presented as means ± SEM. Differences between groups were analyzed using unpaired *t* tests or ANOVA followed by the Student-Newman-Keuls test. DsiRNA, Dicer-substrate siRNA; MCP-1, monocyte chemoattractant protein-1; NGAL, neutrophil gelatinase-associated lipocalin.

To further verify the role of Hmgcs2 in AKI, we knocked down Hmgcs2 in vitro and in vivo. In vitro, under hypoxic stress, knockdown of Hmgcs2 with Dicer-substrate siHmgcs2 increased the expression of NGAL and Bax in cultured normal rat kidney proximal tubular cells (NRK-52E) (Figure 5A and Supplemental Figure 8A). Conversely, after incubating NRK-52E cells with ketone body β-OHB at different dosages, Western blot analysis showed that FADD and NGAL in tubular cells were markedly decreased (Figure 5B). In vivo, knockdown of Hmgcs2 employed the hydrodynamic gene delivery technique, and these mice were then subjected to 1-day ischemic AKI, as depicted in Figure 5, C and D. At 1 day after IRI, knockdown of Hmgcs2 aggravated Scr levels and kidney morphological changes (Figure 5, E and F, and Supplemental Figure 7J). TUNEL staining indicated that knockdown of Hmgcs2 increased the numbers of apoptotic cells in the kidneys compared with vehicle (Figure 5F). The quantitative data were presented in Figure 5G. Western blot assays demonstrated that NGAL and Bax expression was induced in ShHmgcs2 kidneys after IRI, compared with vehicle kidneys (Figure 5, H and I). Meanwhile, knockdown of Hmgcs2 also increased monocyte chemoattractant protein–1 (*MCP-1*), *IL-6*, and *TNF-*α mRNA levels at 1 day after IRI (Figure 5J). Consistently, immunohistochemical staining showed increased CD45^+^ cells in ShHmgcs2 kidneys compared with vehicle kidneys (Figure 5K and Supplemental Figure 7K). These results indicated that Hmgcs2 activation is beneficial in mitigating AKI.

### Knockdown of CNN2 enhances lysine desuccinylation of HMGCS2 in the kidney after AKI.

**Figure 6:**
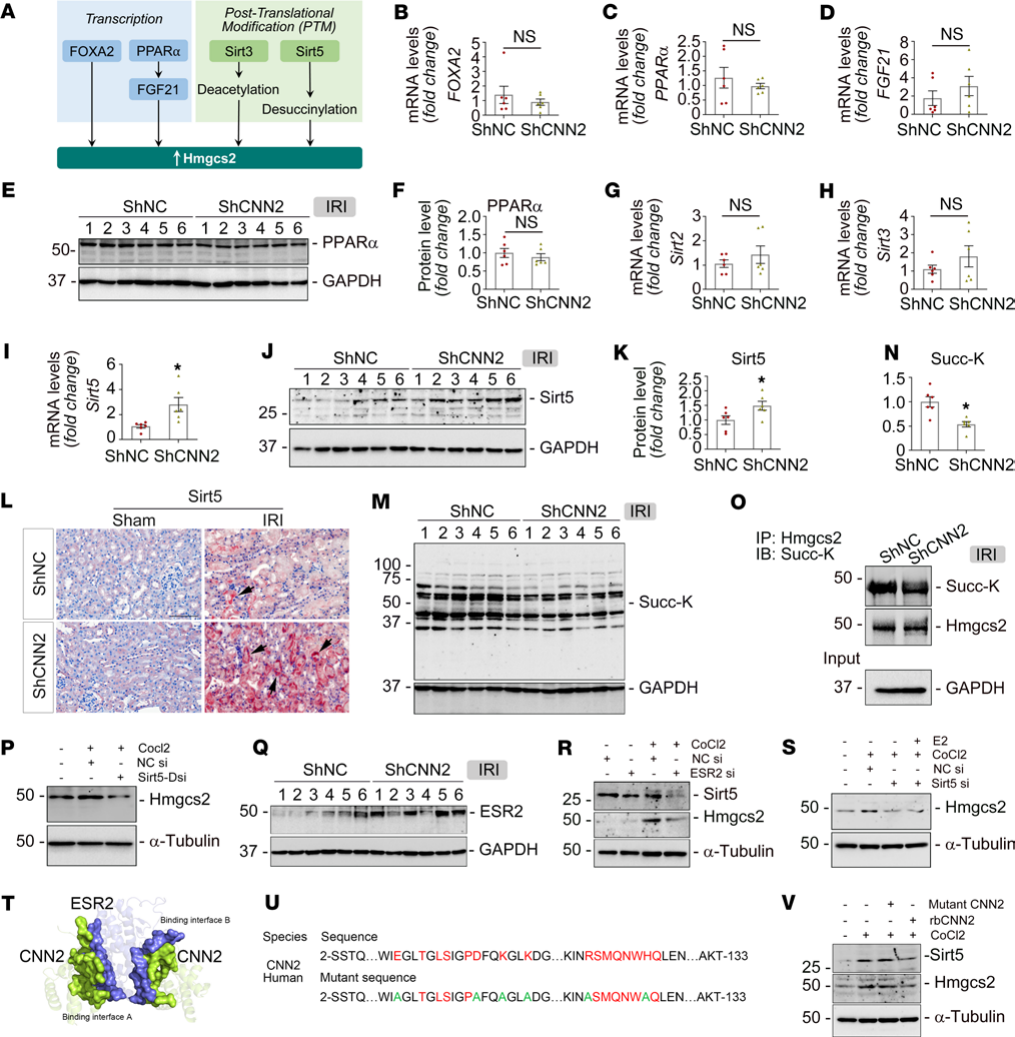
Knockdown of CNN2 enhances lysine desuccinylation of HMGCS2 in the kidney after AKI. (**A**) Schematic diagram. After IRI at 1 day, in ShNC and ShCNN2 mouse kidneys, (**B**–**D**) qPCR analyses revealed FOXA2, PPARα, and FGF21 mRNA levels (*n* = 6). (**E** and **F**) Representative Western blots (**E**) and the quantified data (**F**) of PPARα (*n* = 6). (**G**–**I**) qPCR analyses revealed sirt2, sirt3, and sirt5 mRNA levels. *P < 0.05 (*n* = 6). (**J** and **K**) Representative Western blots (**J**) and the quantified data (**K**) of sirt5. *P < 0.05 (*n* = 6). (**L**) Representative micrographs for sirt5 staining. Scale bar, 50 µm. Arrows indicate the positive staining. (**M** and **N**) Representative Western blot (**M**) and quantified data (**N**) of succinyl-lysine motif (Succ-K). *P < 0.05 (*n* = 6). (**O**) Immunoprecipitation of endogenous Hmgcs2 from the kidney lysates of ShNC and ShCNN2 mice. (**P**) Western blot assay demonstrated that knockdown of sirt5 repressed Hmgcs2 expression in NRK-52E cells under hypoxic stress. (**Q**) Western blot assay demonstrated that knockdown of CNN2 induced ESR2 after IRI. (**R**) Western blot assay demonstrated that knockdown of ESR2 repressed sirt5 and Hmgcs2 expression in NRK-52E cells under hypoxic stress. (**S**) Western blot assay demonstrated that estradiol (100 nM) did not induce Hmgcs2 in NRK-52E cells after knockdown of sirt5, compared with scramble controls. (**T**) Molecular docking analysis showed the binding sites between CNN2 and ESR2. (**U**) The strategy of designing a mutant form of CNN2. (**V**) Western blot assay demonstrated that mutant CNN2 (25 ng/mL) did not affect sirt5 and Hmgcs2 expression in NRK-52E cells, compared with active form of CNN2 human recombinant (rb) protein (25 ng/mL). For Western blot panels, numbers indicate individual animals within each group. Graphs are presented as means ± SEM. Differences between groups were analyzed using unpaired *t* tests.

Next, we sought to determine whether and how CNN2 regulates Hmgcs2 activation after AKI. In general, Hmgcs2 exerts its biological functions mainly through transcription or PTM processes. As illustrated in Figure 6A, 2 transcription factors, forkhead box A2 (FOXA2) and peroxisome proliferator–activated receptor α (PPARα), participate in *Hmgcs2* gene transcription (9). Additionally, Hmgcs2 is posttranslationally modified by mitochondrial sirtuin-3 (sirt3) or sirtuin-5 (sirt5) (15, 19). To explore the mechanisms involved in AKI, we validated the expression of FOXA2, PPARα, fibroblast growth factor 21 (FGF21), sirt2, sirt3, and sirt5. Interestingly, all these genes or proteins showed no differences between ShNC and ShCNN2 kidneys after IRI, except sirt5 (Figure 6, B–H). *Sirt5* mRNA level was increased in ShCNN2 kidneys compared with ShNC after IRI (Figure 6I). Western blot assay showed increased sirt5 protein in ShCNN2 kidneys (Figure 6, J and K). Consistently, immunohistochemical staining showed upregulation of sirt5 in ShCNN2 kidney tubules (Figure 6L). Impressively, knockdown of CNN2 decreased the succinylation status of kidney proteins after AKI (Figure 6, M and N). To elucidate the regulation of sirt5 on Hmgcs2 succinylation in vivo, we immunoprecipitated endogenous Hmgcs2 from vehicle or ShCNN2 kidneys after AKI. Western blot assay demonstrated that Hmgcs2 succinylation was lower in ShCNN2 kidneys compared with ShNC kidneys after AKI (Figure 6O). Interestingly, in vitro, knockdown of sirt5 caused the downregulation of Hmgcs2 in tubular cells (Figure 6P and Supplemental Figure 8B). Because CNN2 physically interacts with ESR2 (5), we assessed ESR2 expression in AKI kidneys. After IRI, ESR2 was induced in ShCNN2 kidneys compared with vehicle kidneys (Figure 6Q). Knockdown of ESR2 in the cultured tubular cells repressed sirt5 and Hmgcs2 induction (Figure 6R and Supplemental Figure 8C). Most importantly, after knockdown of sirt5, estradiol did not induce Hmgcs2 in tubular cells under hypoxic stress (Figure 6S). These data reflected that sirt5 is a key factor in the cascade that connects CNN2 and Hmgcs2. To further determine whether ESR2 is required in a series of effects regulated by CNN2, we designed a mutant form of CNN2. Through a molecular docking study, we identified 2 significant binding sites with extremely low dock energy between CNN2 and ESR2 to form a dense hydrogen bonding network system (Figure 6T and Supplemental Figure 9). Then, we mutated the amino acids with polar charges, including H, R, E, K, and D, and substituted these amino acids with A (Figure 6U). Detailed information is presented in the Supplemental Methods. After treating NRK-52E cells with mutant and regular CNN2 recombinant protein, it was impressive that the mutant CNN2 recombinant protein could not affect sirt5 and Hmgcs2 expression compared with the regular CNN2 recombinant protein (Figure 6V), which suggested that CNN2 influences sirt5 and that Hmgcs2 activities may involve binding to ESR2 at least in the kidney system.

### Knockdown of CNN2 promotes tubular cell survival through desuccinylating Hmgcs2 by Sirt5 in vitro.

**Figure 7:**
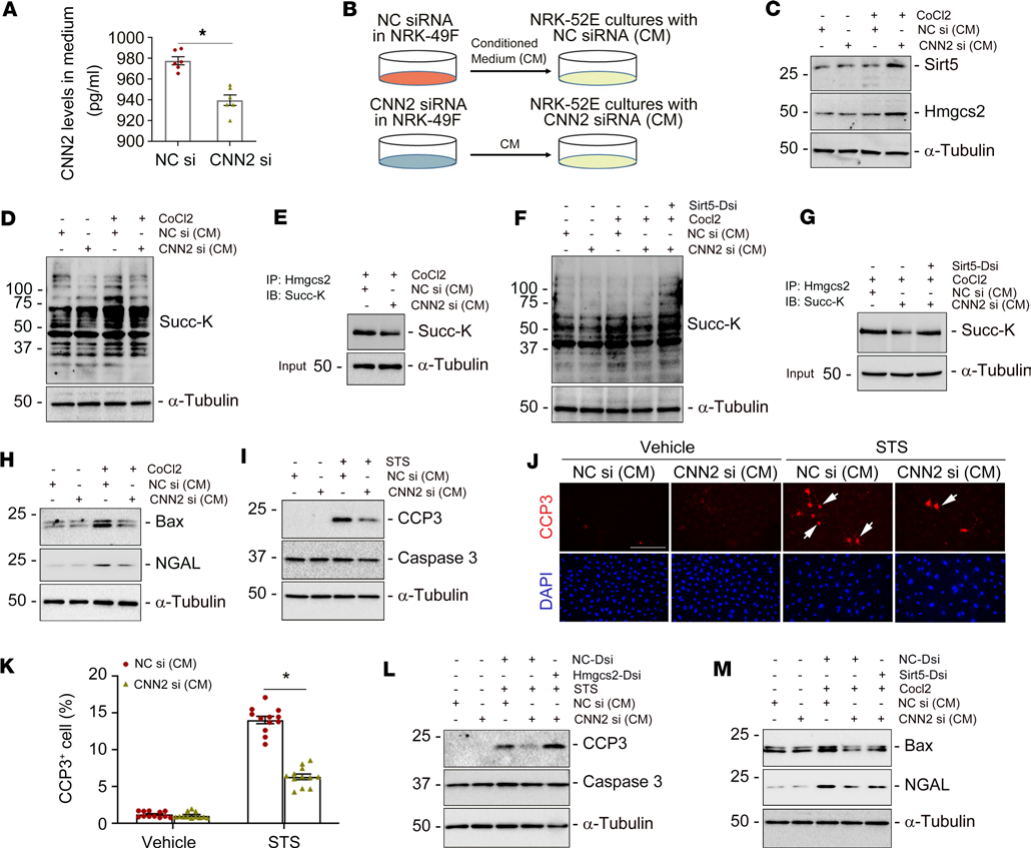
Knockdown of CNN2 promotes Hmgcs2 desuccinylation to repress tubular cell death in vitro. (**A**) ELISA showed CNN2 levels in the conditioned medium (CM) collected from the cultured fibroblasts after knockdown of CNN2 under hypoxic stress. *P < 0.05 (*n* = 6). (**B**) Schematic diagram. (**C**) Western blot assay demonstrated that CNN2-deprived CM enhanced sirt5 and Hmgcs2 expression in NRK-52E cells stimulated with CoCl2 (400 µM). (**D** and **F**) Western blot assay demonstrated CNN2-deprived CM decreased the abundance of succinyl-lysine motif (Succ-K) in NRK-52E cells under hypoxia stress (**D**), but they were increased after knockdown of sirt5 (**F**). (**E** and **G**) Co-immunoprecipitation of endogenous Hmgcs2 from cell lysates of normal control (NC) CM and CNN2-deprived CM. Immunoprecipitation revealed that lysine succinylation on Hmgcs2 is less in CNN2-deprived CM under hypoxia stress (**E**) but increased after knockdown of sirt5 (**G**), compared with controls. (**H**) Western blot assay demonstrated decreased Bax and NGAL levels after incubation with CNN2-deprived CM under hypoxic stress, compared with vehicles. (**I**–**K**) After stimulation with staurosporine (STS, 1 μM) for 3 hours, Western blot assay demonstrated the reduced abundance of cleaved caspase-3 (CCP3) in cultured NRK-52E cells incubated with CNN2-deprived CM (**I**) and immunofluorescence staining showed fewer CCP3^+^ tubular cells after treatment with CNN2-deprived CM (**J**). Quantitative data are presented (**K**). Scale bar, 25 μm. *P < 0.05 (*n* = 3). (**L**) Western blot assay demonstrated that CNN2-deprived CM reduced abundance of CCP3 in cultured NRK-52E cells, but they were increased after knockdown of Hmgcs2, compared with scramble controls. (**M**) Western blot assay demonstrated CNN2-deprived CM repressed Bax and NGAL inductions in cultured NRK-52E cells under hypoxia stress, but they were increased after knockdown of sirt5. Graphs are presented as means ± SEM. Differences between groups were analyzed using unpaired *t* tests or ANOVA followed by the Student-Newman-Keuls test.

To explore how fibroblast-specific CNN2 regulates Hmgcs2 in tubular cells, we transfected normal rat kidney fibroblasts (NRK-49F) with CNN2 siRNA (Supplemental Figure 8D). ELISA showed that CNN2 levels in NRK-49F conditioned medium (CM) were reduced under hypoxic stress after knockdown of CNN2 (Figure 7A). Then, we used CNN2-deprived CM to treat NRK-52E cells (Figure 7B). Western blot assay demonstrated that sirt5 and Hmgcs2 were upregulated in NRK-52E cells after incubation with CNN2-deprived CM under hypoxic stress (Figure 7C). To validate our in vivo findings that knockdown of CNN2 enhanced lysine desuccinylation of Hmgcs2 and accordingly alleviated tubular cell death after IRI, we performed further in vitro studies. First, under hypoxic stress, we examined the level of Hmgcs2 succinylation after incubation with control-CM and CNN2-deprived CM. Western blot assay showed increased protein desuccinylation in tubular cells after incubation with CNN2-deprived CM (Figure 7D). Co-immunoprecipitation demonstrated that Hmgcs2 succinylation was reduced by CNN2-deprived CM (Figure 7E). However, knockdown of sirt5 largely abolished Hmgcs2 desuccinylation in tubular cells (Figure 7, F and G). Then, we examined whether Hmgcs2 desuccinylation could protect tubular cells. As shown in Figure 7H, CNN2-deprived CM markedly reduced Bax and NGAL expression under hypoxic stress. Similarly, CNN2-deprived CM downregulated cleaved caspase-3 in tubular cells after stimulation with a classic apoptosis inducer, staurosporine, as indicated by Western blot assay and immunofluorescence staining (Figure 7, I–K). To provide robust evidence that CNN2 modifies Hmgcs2-mediated ketogenesis to control the fate of tubular cells after AKI, we transfected Dicer-substrate siHmgcs2 to knock down Hmgcs2 in NRK-52E cells. Western blot assays demonstrated that, under hypoxic stress, knockdown of Hmgcs2 or sirt5 largely abolished the beneficial effects on tubular cell death by CNN2-deprived CM (Figure 7, L and M).

**Figure 8:**
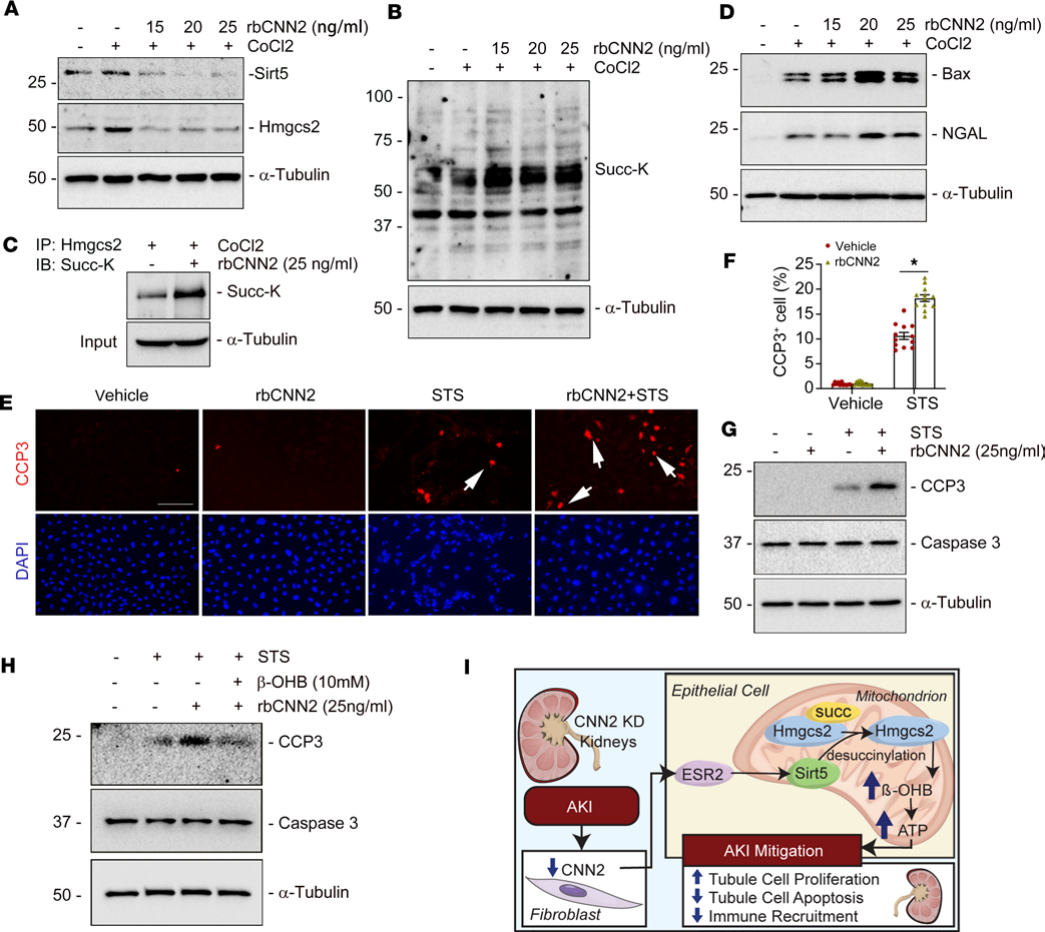
CNN2 inhibits Hmgcs2 desuccinylation to accelerate tubular cell death in vitro. (**A**) Representative Western blots showed that human CNN2 recombinant protein markedly repressed sirt5 and Hmgcs2 expression in NRK-52E cells at different dosages under hypoxic stress. (**B**) Western blot assay demonstrated CNN2 recombinant protein enhanced the abundance of Succ-K in NRK-52E cells at different dosages under hypoxia stress, compared with controls. (**C**) Co-immunoprecipitation revealed that lysine succinylation on Hmgcs2 is increased after human CNN2 recombinant protein treatment under hypoxia stress. (**D**) Western blot assay showed that human CNN2 recombinant protein induced Bax and NGAL expression at different dosages under hypoxic stress, compared with vehicle. (**E**–**G**) After stimulation with staurosporine (1 μM) for 3 hours, immunofluorescence staining showed increased CCP3^+^ tubular cells after being incubated with human CNN2 recombinant protein (**E**) and quantitative data are presented (**F**). Scale bar, 25 μm. *P < 0.05 (*n* = 3). Western blots demonstrated the upregulated abundance of CCP3 in cultured NRK-52E cells after incubation with CNN2 recombinant protein (**G**). (**H**) Western blot assay showed β-OHB decreased the abundance of CCP3 after being treated with CNN2 recombinant protein in cultured NRK-52E cells. (**I**) Schematic diagram depicts knockdown of CNN2 enhanced ketogenesis to mitigate AKI. Graphs are presented as means ± SEM. Differences between groups were analyzed using ANOVA followed by the Student-Newman-Keuls test.

Further, we applied human CNN2 recombinant protein to treat tubular cells at different dosages. Western blot assay demonstrated that compared with CNN2-deprived CM, CNN2 protein repressed sirt5 and Hmgcs2 under hypoxic stress (Figure 8A). Co-immunoprecipitation showed that CNN2 recombinant protein increased lysine succinylation of Hmgcs2 in tubular cells (Figure 8, B and C). Impressively, CNN2 protein induced tubular cell apoptosis under hypoxic stress, as shown by Western blot assay and immunostaining (Figure 8, D–G). To restore tubular cell injury, we further treated tubular cells with β-OHB. We found that β-OHB inhibited CNN2 protein–induced tubular cell apoptosis, as evidenced by reduced cleaved caspase-3 after stimulation with staurosporine (Figure 8H). Thus, we conclude that knockdown of CNN2 enhances endogenous ketogenesis by desuccinylating Hmgcs2 through a series of reactions, which provides more energy for AKI mitigation and repair (Figure 8I).

## Discussion

Energy metabolism, such as glucose, fatty acid, and amino acid metabolism, is an indispensable process for maintaining cell homeostasis. Research over the past several decades has expanded our knowledge of energy metabolism in many disease-oriented research fields, such as diabetes, obesity, cardiology, and cancer. This metabolic process has evolved to have multiple pathways that respond to various pathologic stresses by generating energy. In recent years, along with the reported beneficial effects of sodium-glucose cotransporter-2 inhibitor treatment on kidneys (20, 21), elucidating the pathogenesis of kidney disease from the perspective of ketogenesis has been increasingly appreciated.

In this study, we report that CNN2, a prominent actin stabilizer, was predominantly induced in fibroblasts/pericytes after AKI (Figure 1), and it plays a key role in forming a favorable KLM. In AKI, fibroblast activation is an early event. Upon injury, fibroblasts are immediately activated and send out “SOS” signals to neighboring cells in the KLM to rescue the kidney (6). In structure, fibroblasts physically contact tubular cells, which generates forces important for creating the KLM. Earlier studies demonstrated that deleting CNN2 attenuates calcific aortic valve disease by reducing myofibroblast differentiation (22), and deleting CNN2 in fibroblasts further increases myosin II–dependent cell traction force (23). In the kidney system, knockdown of CNN2 caused early activation of fibroblasts after AKI (Figure 3). These findings reflect the capacities of CNN2 in mediating mechanical forces. However, such forces in the KLM also depend on the extracellular matrix and the fluids that surround cells (24) because the formed KLM influences biochemical and protein compositions that regulate cell behavior. Interestingly, CNN2 is detectable and elevated in the circulation of patients with CKD (5). Serum CNN2 has even been used as a surrogate marker for the diagnosis of tubal ectopic pregnancy (25). Furthermore, CNN2 is also partially expressed by some non–smooth muscle cells, such as epithelial cells. It can translocate to damaged lysosomes caused by lysosomal membrane permeabilization (LMP) and is ubiquitylated for timely dissociation from lysosomes during lysophagy after various injuries (26), and LMP often contributes to tubular cell death and inflammation (27). Therefore, among all components of KLM, CNN2 plays a unique role by serving as a biochemical effector or a mediator of mechanotransduction. Besides, CNN2 is reported to regulate macrophage activities in various diseased scenarios (28–30), and our study also showed that knockdown of CNN2 reduced F4/80^+^ macrophages in the ischemic kidney (Figure 3). Given its role in the KLM, it is unsurprising that knockdown of CNN2 preserved kidney function, reduced cell death and inflammation, mitigated AKI, and improved the long-term kidney outcomes (Figures 2 and 3 and Supplemental Figures 2 and 3).

In CKD, CNN2 interacts with ESR2 and subsequently activates tubular PPARα to alleviate kidney fibrosis (5). ESR2 belongs to the superfamily of nuclear hormone receptors that function as transcription factors. It has been shown to localize to mitochondria in a ligand-dependent or -independent manner and can stimulate mitochondrial metabolism to meet energy needs for determining cell behavior (31). PPARα is a regulator of the FAO pathway (5, 32). However, different from CKD, it is surprising that CNN2 knockdown has minor effects on the changes of PPARα and the key enzymes in the FAO pathway after AKI (Figures 4 and 6). Therefore, we tested if any alternative fuel sources are generated for AKI mitigation in the current model. Our proteomics unbiasedly identified that Hmgcs2 was markedly induced in the AKI kidney proximal tubules after CNN2 knockdown (Figure 4). Hmgcs2 is the rate-limiting enzyme responsible for regulating ketogenesis to produce ketone bodies. In general, it is constitutively expressed by the liver. However, our results show that CNN2 knockdown has little effect on liver Hmgcs2 expression (Figure 4). But, β-OHB, the most abundant circulating ketone body, was increased in the blood of ShCNN2 mice after AKI (Figure 4). β-OHB can prime the epigenetic forkhead transcription factor O3–dependent pyroptotic pathway to attenuate kidney ischemic injury (12). Furthermore, knockdown of Hmgcs2 aggravated ischemic AKI (Figure 5), consistent with the results generated using Hmgcs2 genetic mouse models from other groups (33). These results reflect that the kidney might be ketogenic under AKI stress. Generating ketone bodies might not be limited to fasting and diabetic conditions (34), although a recent study reported that kidney Hmgcs2 does not contribute to circulating ketones during fasting (35). From the perspective of cellular localization, Hmgcs2 is specifically expressed in proximal tubules (Figure 4). Its functions, other than systemic ketone provision, likely include antiinflammation and anti–oxidative stress (36, 37). In circumstances where damaged proximal tubules are unable to fully recover, it could cause a metabolic switch along with AKI progression (38). It is also important to note that the overproduction of ketone bodies via ketogenesis can cause problems due to their acidic nature, so any therapies inducing ketogenesis must be carefully balanced.

After AKI, how does ESR2 interact with Hmgcs2 in the kidney? Amid ketone body synthesis, mitochondrial Hmgcs2 condenses acetyl-CoA and acetoacetyl-CoA into HMG-CoA and is transcriptionally regulated by FOXA2, PPARα, and FGF21 or is posttranslationally modified by sirtuins, a class of proteins that possess either mono-ADP-ribosyltransferase or deacylase activity (9). Our study revealed that sirt5 was upregulated in ShCNN2 kidneys after AKI but that other sirtuins were not. Knockdown of ESR2 repressed sirt5 and Hmgcs2 activities in tubular cells, whereas exogenous ESR2 had no effects on Hmgcs2 if sirt5 was knocked down (Figure 5). These results indicate that mitochondrial sirt5 is a potential bridge connecting ESR2 with HMGCS2. In particular, ESR2 is required by CNN2 to regulate these downstream biological processes (Figure 6 and Supplemental Figure 9). Sirtuins have been reported to regulate steroid hormone signals, including estrogen receptors, through various molecular mechanisms; for instance, sirt1 serves as an ESR1 coactivator (39). Clearly, the relationship between ESR2 and Hmgcs2 needs further investigation.

Furthermore, our study reports an interesting PTM that occurs in the kidney amid AKI repair. PTMs are covalent processing events that increase the functional diversity of the proteome by proteolytic cleaving of regulatory subunits, adding functional groups to amino acids, or degrading entire proteins (40). Sirtuins are the members using coenzyme nicotinamide adenine dinucleotide to deacetylate lysine residues in histone and nonhistone proteins to participate in numerous biological processes such as DNA repair and mitochondrial energy homeostasis (41). There are 7 mammalian sirtuins that regulate the acylation of many pathways, including 3 nuclear (sirt1, 6, and 7), 1 cytoplasmic (sirt2), and 3 mitochondrial sirtuins (sirt3, 4, and 5) (42). After AKI, interestingly, Hmgcs2 was activated in ShCNN2 kidneys through sirt5-mediated desuccinylation but not sirt3-mediated deacetylation for AKI mitigation (Figures 6–8). Sirt5 was initially identified as a deacetylase targeting carbamoyl phosphate synthetase to regulate the urea cycle in the liver (43). However, it has been discovered that sirt5 functions as a demalonylase, deglutarylase, and desuccinylase rather than as a deacetylase. It removes succinyl, malonyl, and glutaryl moieties from target lysines within the mitochondrial matrix and other subcellular compartments (44). Sirt5 can repress biochemical activity and cellular respiration through pyruvate dehydrogenase complex and succinate dehydrogenase (45). In the liver, several metabolic pathways, including β-oxidation and ketogenesis, are hypersuccinylated in sirt5^–/–^ animals (19). Loss of sirt5 leads to decreased β-OHB production (19). Although its role in AKI is relatively controversial (46–49), sirt5 still serves as a global regulator of lysine succinylation in mitochondria and mediates ketogenesis through Hmgcs2. Of note, sirt3 is responsible for global protein deacetylation in mitochondria. It also deacetylates Hmgcs2 and regulates ketone body production (15), but knockdown of CNN2 has not affected its regulation after AKI. Therefore, further exploration is needed to illustrate the role of actin stabilizers in regulating PTMs in the kidney.

Our study has some limitations. First, although we provided robust evidence to show the cell type specificity that expresses CNN2, lacking genetic mouse models still somewhat limited our interpretation of CNN2 functions in AKI. Second, female mice are relatively resistant to IRI, so we only used male mice in this study. Third, since non–smooth muscle cells such as epithelial cells also express CNN2, it remains unclear whether CNN2 directly regulates Hmgcs2 in kidney tubules after AKI. Cell type–specific knockouts should be explored to pinpoint the location of CNN2-mediated Hmgcs2 modulation further.

In summary, our study described a comprehensive process of how CNN2 constructs a metabolic microenvironment through posttranslationally modifying Hmgcs2 to dictate the outcomes of AKI. In this microenvironment, beyond serving as metabolic substrates, kidney ketone bodies are vital metabolic and signaling mediators for AKI repair. Further dissecting energy metabolism in actin stabilizer-mediated microenvironment formation is believed to complement a repertoire of known therapeutic options for preventing or mitigating AKI in the future.

## Methods

The detailed information of methodology, usage of chemical and biological reagents, antibodies, and nucleotide sequences of the primers is presented in the Supplemental Methods.

### Statistics.

All data were expressed as mean ± SEM if not specified otherwise in the legends. Statistical analysis of the data was performed using GraphPad Prism 9. Comparison between 2 groups was made using a 2-tailed Student’s *t* test or the rank-sum test if data failed a normality test. Statistical significance for multiple groups was assessed by 1-way ANOVA, followed by the Student-Newman-Keuls test. Results are presented in dot plots, with dots denoting individual values.

### Study approval.

All animal experiments were performed in accordance with institutional and federal guidelines and approved by the Institutional Animal Care Committee of the University of Connecticut School of Medicine (Protocol number: AP-200105-0923). All patients included in the presented study had signed the informed consent forms before they underwent kidney biopsy or nephrectomy. All studies involving human kidney sections were approved by the Institutional Review Board at the University of Pittsburgh and the University of Connecticut School of Medicine.

### Data availability.

Data are available in the Supporting Data Values XLS file. Raw mass spectrometry data were deposited in MassIVE with the data set identifier MSV000090190. The bulk RNA-sequencing data were deposited in the Gene Expression Omnibus under accession number GSE226534.

## Author contributions

YG and DZ conceived the project. YG and DZ wrote and revised the manuscript. YG, ZP, PG, and Yuanyuan Wang performed most in vivo, ex vivo, and in vitro studies involving mRNA expression analysis; Western blotting assay; immunostaining; and imaging. YG, HL, YP, and SL performed bioinformatic analysis. YG, GH, DLK, Yanlin Wang, SIB, YL, and DZ edited the manuscript. DZ supervised the entire project.

## Supplementary Material

Supplemental data

Supporting data values

## Version 1 09/26/2023

In-Press Preview

## Version 2 11/08/2023

Electronic publication
