# Supplementary material for: Calponin 2 regulates ketogenesis to mitigate acute kidney injury
Source: JCI Insight. 2023 Nov 8;8(21):e170521. doi: 10.1172/jci.insight.170521 (PMC10721266; doi:10.1172/jci.insight.170521)
Supplement: Supplemental data [file jciinsight-8-170521-s212.pdf]

Calponin 2 regulates ketogenesis to mitigate acute kidney injury

Yuan Gui[1], Zachary Palanza[1], Priya Gupta[1], Hanwen Li[2], Yuchen Pan[3], Yuanyuan Wang[1], Geneva Hargis[4], Donald L. Kreutzer[5], Yanlin Wang[1], Sheldon I. Bastacky[6], Yansheng Liu[7,8], Silvia Liu[6], Dong Zhou[1]

Supplementary Materials

Detailed Methods

Public data mining

Public database Tabula Sapiens, a multiple-organ, single-cell transcriptomic atlas of humans, was mined in the study.[S1] Per organ, single cell gene count matrix was downloaded and loaded into R package Seurat for further analysis.[S2] CNNs expression ratio was calculated by the number of cells with CNN2 expression over the total number of cells.

Another public single-cell nucleus RNA-seq study on mouse acute kidney injury was explored in this project.[S3] For time point control, 4h, 12h and 48h, single cell gene count matrix was downloaded from GEO database by accession ID GSE139107.[S3] Multiple libraries were integrated and normalized by R package Seurat.[S2] Cell clustering was performed and cell population was annotated based on the gene signatures in the reference paper.[S3] Eventually, CNN2 expression was visualized per cell population by Seurat.

Bulk-tissue RNA sequencing and bioinformatics analysis

The mouse kidney tissues subjected to bulk RNA sequencing were collected at day 0, 1, and 3 after ischemic AKI. The Mouse samples sequencing was performed using Illumina HiSeqX platform (MedGenome Inc., Foster City, CA). TruSeq Stranded mRNA library preparation kit was used to perform RNA sequencing. Quality control and trimming was performed on the raw RNA-seq reads by tool Trimmomatic to filter out reads with low sequencing quality.[S4] Then the surviving reads were aligned to mouse reference genome

mm10 and quantified by STAR aligner.[S5] Based on the gene by sample count matrix, normalization and differential expression analysis was performed by R package DESeq2.[S6] The CNN2 gene expression figure was visualized by R package ggplot2.[S7]

Mouse models of acute kidney injury (AKI)

Male Balb/c mice weighing approximately 20-22g were obtained from the Jackson Laboratory (Bar Harbor, ME). Two mouse AKI models were constructed by employing renal ischemia-reperfusion injury (IRI) and cisplatin (Cis) injection, respectively. For the IRI model, in brief, bilateral renal arteries were clipped for 25 minutes (moderate AKI) or 30 minutes (severe AKI) using microaneurysm clamps. The mouse body temperature was maintained between 36°C and 37.5°C by using a temperature-controlled heating system during the ischemic period. Mice were sacrificed 1 or 7 days after IRI. The cisplatin-induced AKI model involved a single intraperitoneal injection of cisplatin at 30 mg/kg body weight, and then mice were sacrificed 3 days after injection. Serum and kidney samples were collected for further analyses.

Customized short hairpin RNA (ShRNA) specific for the mouse CNN2 gene was designed by Longqian Biotech. The target sequence of the murine CNN2 is CTCCAACTTCATCAAGGCCAT (position in CNN2 mRNA 342-362). The target sequence of the scrambled ShRNA is CAACAAGATGAAGAGCACCAA. The mouse CNN2-specific ShRNA was subcloned into pLKO.1 vector. The promoter is U6. The empty vector and ShCNN2 were administered to mice by a hydrodynamic-based gene transfer technique through rapid injection of a large volume of solution through the tail vein as described elsewhere.[S8] In a separate experiment, male Balb/c mice were administrated with ShHmgcs2 (designed by Longqian Biotech). Briefly, ShCNN2 (1mg/kg) or ShHmgcs2 (1mg/kg) was diluted in 1.8 ml saline and injected through the tail vein into mouse circulation within 5-10 seconds. Mice from the vehicle group were identically injected with an empty vector. In all models, ShCNN2 or ShHmgcs2 was administered 7 days and 1 day before bilateral IRI or cisplatin injection, respectively. All proposed animal experiments were approved by the Institutional Animal Care and Use Committee at the University of Connecticut, School of Medicine.

Human Kidney Biopsy Specimens

Human kidney specimens were obtained from diagnostic renal biopsies performed at the Presbyterian Hospital of the University of Pittsburgh Medical Center. Nontumor kidney tissue from the patients who had renal cell carcinoma and underwent nephrectomy was used as normal controls. All patients included in the presented study have signed the informed consent forms before they underwent kidney biopsy or nephrectomy. All studies involving human kidney sections were approved by the Institutional Review Board at the University of Pittsburgh and the University of Connecticut, School of Medicine.

Determination of Serum Creatinine and blood urea nitrogen

Blood urea nitrogen (BUN) and serum creatinine (Scr) levels were determined using the QuantiChrom™ Urea (DIUR-100) and Creatinine (DICT-500) assay kits, according to the protocols specified by the manufacturer (BioAssay Systems, Hayward, CA). The levels of BUN and Scr were expressed as milligrams per 100 mL (dL).

β-Hydroxybutyrate (β-OHB) Measurement

The levels of serum β-OHB were measured by using β-Hydroxybutyrate Assay Kit (MAK41-1KT, Sigma-Aldrich) according to the manufacturer's instructions.

ATP Measurement

ATP content in kidney tissue was measured by using the ATP Colorimetric/Fluorometric Assay Kit (K354-100, BioVision, Waltham, MA), according to the manufacturer’s instructions. Data were normalized for total protein content.

Alanine Transaminase (ALT) Measurement

The levels of ALT in blood were measured by using the ALT Assay Kit (EALT-100, BioAssay Systems, Hayward, CA), according to the manufacturer's instructions.

Enzyme-linked immunosorbent assay (ELISA)

The rat CNN2 (MBS075726) Elisa kits were purchased from MyBioSource, Inc (San Diego, CA). This assay employs the quantitative sandwich enzyme immunoassay technique. Antibody specific for CNN2 has been pre-coated onto a 96-well strip plate. We prepared 7 wells for standard, 1 well for blank and added 100 µl each of standard, blank, and sample dilutions into the appropriate wells. The plate was then incubated at 37°C for 2 hours. Liquid from each well was removed and 100µl of Detection Reagent A working solution was added to each well. The plate was incubated again at 37°C for 1 hour. After washing, 100µL of Detection Reagent B working solution was added to each well and the plate was incubated for 30 minutes at 37°C. Following a wash to remove any unbound reagent, 90 µl of substrate solution was added to the wells, and color developed in proportion to the amount of CNN2 bound in the initial step. The color development was stopped, and the intensity of the color was measured immediately using a microplate reader set to 450 nm.

Quantitative Real-Time Reverse Transcription PCR (qPCR)

Total RNA isolation and qPCR were carried out by procedures described previously.[S9] Briefly, the first strand cDNA synthesis was carried out using a reverse transcription system kit according to the instructions of the manufacturer (Bio-Rad). qPCR was performed on an ABI PRISM 7000 sequence detection system (Applied Biosystems, Foster City, CA). The mRNA levels of various genes were calculated after normalizing with β-actin. Primer sequences used for amplifications are presented in Supplementary Table S1.

Histology and Immunohistochemical Staining

Paraffin-embedded human kidney biopsy sections (2.5 μm thickness) and mouse kidney sections (3 μm thickness) were prepared by a routine procedure. The sections were stained with periodic acid–Schiff staining reagents by standard protocol. Immunohistochemical staining was performed according to the established protocol as described previously.[S9] After incubation with primary antibodies at 4°C overnight, the slides were then stained with HRP-conjugated secondary antibody (Jackson ImmunoResearch Laboratories, West Grove, PA). Non-immune normal IgG was used to replace primary antibodies as a negative control, and no staining was visible. Slides were viewed under an Olympus BX43 microscope equipped with a digital camera (Allentown, PA). The detailed information of the applied primary and secondary antibodies was presented in Supplementary Table S2.

Immunofluorescence staining

Kidney cryosections or cultured cells were fixed with 3.7% paraformaldehyde for 15 min at room temperature and immersed in 0.2% Triton X-100 for 10 min. After blocking with 10% donkey serum for 1 hour, the slides were immunostained with primary antibodies against cleavage-caspase 3 or double immunostained with anti-CNN2 and PDGFRβ, F4/80, and CD31 or HMGCS2 and AQP1. These slides were then stained with Cy2- or Cy3-conjugated secondary antibody (Jackson ImmunoResearch Laboratories, West Grove, PA). Slides were viewed under ZEISS LSM 900 confocal microscope equipped with a digital camera (Dublin, CA). The detailed information of the applied primary and secondary antibodies was presented in Supplementary Table S2.

Detection of apoptotic cells

Apoptotic cell death was determined by using TUNEL staining with a DeadEnd Fluorometric Apoptosis Detection System (Promega, Madison, WI) or immunostaining with anti-cleaved caspase 3 (#9664S, Cell Signaling Technology, Danvers, MA).

Co-immunoprecipitation

Co-immunoprecipitation was carried out using an established method. Briefly, kidney tissues or NRK-52E cells stimulated with CoCl2 were lysed on ice in 1 ml non-denaturing lysis buffer that contained 1% Triton X-100, 0.01 mol/l Tris-HCl (pH 8.0), 0.14 mol/l NaCl, 0.025% NaN3, 1% protease inhibitors cocktail, and 1% phosphatase inhibitors cocktail I and II (Sigma). Kidney tissues or cells lysates were incubated overnight at 4°C with 2 mg of anti-Hmgcs2 (sc-393256), followed by precipitation with 100 ml of protein A/G Plus-agarose for 3h at 4°C. The precipitated complexes were separated by SDS–polyacrylamide gel electrophoresis and immunoblotted with specific antibodies against succinyllysine (PTM-401, PTM Biolabs, Chicago, IL) or Hmgcs2 (LS-B11023, LsBio, Seattle, WA), respectively.

Western Blot Analysis

Kidney tissues were lysed with radioimmune precipitation assay (RIPA) buffer containing 1% NP-40, 0.1% SDS, 100 μg/ml PMSF, 1% protease inhibitor cocktail, and 1% phosphatase I and II inhibitor cocktail (Sigma) in PBS on ice. The supernatants were collected after centrifugation at 13,000×g at 4°C for 15 min. Protein expression was analyzed by western blot analysis as described previously. The detailed information of the applied primary and secondary antibodies was presented in Supplementary Table S2.

Global Proteomics Sample Preparation

Kidney tissues were processed for proteomics following a shotgun approach published previously.[S10] In brief, ShNC and ShCNN2 mice kidneys were lysed using SDS buffer (4% SDS, 50mM EDTA, 20mM DTT, 2% Tween 20, 100mM Tris-HCl, pH 8.0) and sonication (Misonix Sonicator 3000 Ultrasonic Cell Disruptor). The proteins were processed following the suspension trapping (STrap) digestion protocol using in-house packed filters (GF/F, Whatman). The peptides were desalted using the spinnable StageTip protocol and stored under -80°C until further analysis.

LC-MS/MS analysis

The LC-MS/MS analysis was performed using an Orbitrap Eclipse MS (Thermo Scientific) coupled with an Ultimate 3000 nanoLC system and a FAIMS Pro Interface (Thermo Scientific). Peptides were first loaded onto a trap column and then separated by an analytical column (PepMap C18, 2.0 um; 15 cm x 75 mm I.D.; Thermo Scientific) at a 250 nl/min flow rate using a binary buffer system (buffer A, 0.1% formic acid in water; buffer B, 0.1% formic acid in acetonitrile) with a 165-min gradient (1% to 10% in 8 min; then to 25% buffer B over 117 min; 25% to 32% buffer B in 10 min, then to 95% buffer B over 3 min; back to 1% B in 5 min, and stay equilibration at 1% B for 20 min). Multiple CVs (-40, -60, and -80) were applied for FAIMS separation. For all MS experiments, the survey scans (MS1) were acquired at a resolution of 60,000 in the Orbitrap. The maximum injection time was set to "Auto," and AGC target was set to "Standard." Monoisotopic peak selection was set to "Peptides," and the charge state filter was set to 2-7. For MS/MS acquisition, precursors were isolated with a width of 1.6 m/z, fragmented with HCD using 30% collision energy with a Dynamic maximum injection time, and collected in Orbitrap at 15,000 resolutions. The dynamic exclusion was set to 30 s and can be shared across different FAIMS experiments.

Protein identification and quantitation

Protein quantitation was performed using the MaxQuant software (version 2.1.3.0) with most of the default parameters, including trypsin as an enzyme with a maximum of two missed cleavage sites; acetylation (protein N-terminal and Lys) and oxidation (Met) as variable modifications; cysteine carbamidomethylation as a fixed modification; peptide length must be at least 7 amino acids; false discovery rate (FDR) was set to 1% for both protein and peptide identification. The MaxQuant output (proteinGroups.txt) was log2-transformed prior to downstream data analyses such as missing value imputation, Hierarchical clustering, Principal Component Analysis (PCA), t-tests, correlation, and volcano plots, which were performed in the Perseus environment using default parameters (version 1.6.2.3). Gene Ontology (GO) and Kyoto Encyclopedia of Genes and Genomes (KEGG) pathway analyses were performed using DAVID Bioinformatics Resources 6.8 (https://david.ncifcrf.gov/home.jsp).

Molecular docking study

Amino acid sequences for human Calponin-2 (CNN2) and Estrogen receptor beta (ESR2) were retrieved from the UniProt databank with ID Q99439 and Q92731, respectively. The protein structure of CNN2 and ESR2 were both obtained from the RCSB PDB databank (PDB ID: 1WYN and 1L2J). Water molecules, co-crystallized ligand and ions in protein complex were removed for further docking process. The interaction region were limited to ERK-signaling binding region (residues number: 29-129) for CNN2, and Ligand binding region (LBD) for ESR2 protein.

The following three steps (blind docking and precise docking) were implanted to explore possible binding interface between CNN2 and ESR2. Docking simulations were performed with Rosetta software.[S11] The docking protocol began with a rigid-body blind docking in which all heavy atoms of protein ESR2 and CNN2 were strictly position restrained. In this step, we randomly place the proteins roughly facing each other, and at least 2000 docking conformations were obtained for further analysis. The rigid docking conformations were clustered by using root-mean-square deviation (RMSD) values, and finally clustered position with highest top 2 ranking ratio were selected as potential binding spot. Binding spots far away from the others were manually deleted. Using this approach, we were able to achieve an acceptable-quality prediction for CNN2 “binding pocket” of ESR2. Rosetta software is most accurate when docking locally. In precise docking, the aptamer was randomly placed (within ~10 Å) within the binding pockets, and then the input structure of aptamer was perturbed by 3 Å translation and 8° rotation before the start of every individual simulation. During precise docking, the side chains of the protein residues at the binding pocket and the complete CNN2 were allowed to move. Finally, a maximum number of 100 conformers were considered, and the conformation with the lowest binding energy in two binding spots were selected for final molecular dynamics simulations.

In order to check the stability of CNN2-ESR2 complex, the best docking result in each binding spots were employed for molecular dynamics (MD) simulations. It was carried out by AMBER software (version 16), using AMBER ff99SB force field for complex.[S12] Hydrogen atoms were added to the initial CNN2-ESR2 complex model using the leap module, setting ionizable residues as their default protonation states at a

neutral PH value. The complexes were both solvated in a cubic periodic box of explicit TIP3P water model that extended a minimum 10 Å distance from the box surface to any atom of the solute. The particle mesh Ewald (PME) method for simulation of periodic boundaries was used to estimate the long-range electrostatic interactions with a cutoff of 10.0 Å. All bond lengths were constrained using the SHAKE algorithm and integration time step was set to 2fs using the Varlet leapfrog algorithm. To eliminate possible bumps between the solute and the solvent, the entire systems was minimized in two steps. Firstly, the complex was restrained with a harmonic potential of the form k (Δx)2 with a force constant k =100 kcal/mol-1Å-2. The water molecules and counter ions were optimized using the steepest descent method of 2500 steps, followed by the conjugate gradient method for 2500 steps. Secondly, the entire system was optimized by using the first step method without any constraint. These two minimization steps were followed by annealing simulation with a weak restraint (k=100kcal/mol-1Å-2) for the complex and the entire system was heated gradually in the NVT ensemble from 0 to 298K over 500ps. After the heating phase, a 10ns MD simulation was performed under 1atm. The constant temperature was selected at 298K with the NPT ensemble. Constant temperature was maintained using the Langevin thermostat with a collision frequency of 2ps-1. The constant pressure was maintained employing isotropic position scaling algorithm with a relaxation time of 2ps. RMSD values were tested to monitor the conformation fluctuations of CNN2-ESR2 complex. Based on the final 10ns MDs trajectory, 3000 snapshots were extracted from the last 3ns trajectory for the final average structure of complex.

Cell culture and treatment

Normal rat kidney fibroblasts (NRK-49F) and normal rat kidney epithelial cells (NRK-52E) were obtained from the American Type Culture Collection (ATCC, Manassas, VA). For conditioned media (CM) collections, under CoCl2-induced hypoxic stress, we first transferred CNN2 siRNA (4390815, ThermoFisher Scientific, Waltham, MA) into cultured NRK-49F for 24 hours and then cultured with serum-free medium. After additional 24 hours. cultural medium was harvested and subjected to centrifugation

(3000 rpm for 10 min at 4 °C). The conditioned medium (CM) was aliquoted and stored at -80°C for subsequent experiments. Serum-starved NRK-52E cells were then transfected with sirt5-Dicer siRNA (rn.Ri.sirt5.13, IDT, Coralville, Iowa), Hmgcs2-Dsicer siRNA (rn.Ri.Hmgcs2.13, IDT, Coralville, Iowa), or ESR2-Dsicer siRNA (hs.Ri.ESR2.13, IDT, Coralville, Iowa) or treated with the CM or CNN2 human recombinant protein (ab177711, abcam, Waltham, MA) or mutant CNN2 human recombinant protein (synthesized by MyBiosource, San Diego, CA) or β-OHB (Sigma-Aldrich, St. Louis, MO) or estradiol (HY-B0141, MedChemExpress, NJ, USA) after stimulated with staurosporine (S4400) or CoCl2 (232696; Sigma, St Louis, MO) stress. All in vitro experiments were repeated at least three times.

Statistics

All data were expressed as mean ± SEM if not specified otherwise in the legends. Statistical analysis of the data was performed using GraphPad Prism 9 (GraphPad Software, San Diego, CA). Comparison between two groups was made using a two-tailed Student's t-test or the Rank Sum Test if data failed a normality test. Statistical significance for multiple groups was assessed by one-way or two-way ANOVA, followed by the Student-Newman-Keuls test. Results are presented in dot plots, with dots denoting individual values. $P < 0.05$ was considered statistically significant.

References

S1 Tabula Sapiens, C. *et al.* The Tabula Sapiens: A multiple-organ, single-cell transcriptomic atlas of humans. *Science* **376**, eabl4896, doi:10.1126/science.abl4896 (2022).

S2 Hao, Y. *et al.* Integrated analysis of multimodal single-cell data. *Cell* **184**, 3573-3587 e3529, doi:10.1016/j.cell.2021.04.048 (2021).

S3 Kirita, Y., Wu, H., Uchimura, K., Wilson, P. C. & Humphreys, B. D. Cell profiling of mouse acute kidney injury reveals conserved cellular responses to injury. *Proc Natl Acad Sci U S A* **117**, 15874-15883, doi:10.1073/pnas.2005477117 (2020).

S4 Bolger, A. M., Lohse, M. & Usadel, B. Trimmomatic: a flexible trimmer for Illumina sequence data. *Bioinformatics* **30**, 2114-2120, doi:10.1093/bioinformatics/btu170 (2014).

S5 Dobin, A. *et al.* STAR: ultrafast universal RNA-seq aligner. *Bioinformatics* **29**, 15-21, doi:10.1093/bioinformatics/bts635 (2013).

S6 Love, M. I., Huber, W. & Anders, S. Moderated estimation of fold change and dispersion for RNA-seq data with DESeq2. *Genome Biol* **15**, 550, doi:10.1186/s13059-014-0550-8 (2014).

S7 H, W. *ggplot2: Elegant Graphics for Data Analysis*. (Springer-Verlag, 2016).

S8 Gui, Y. *et al.* Calponin 2 harnesses metabolic reprogramming to determine kidney fibrosis. *Mol Metab* **71**, 101712, doi:10.1016/j.molmet.2023.101712 (2023).

S9 Fu, H. *et al.* The hepatocyte growth factor/c-met pathway is a key determinant of the fibrotic kidney local microenvironment. *iScience* **24**, 103112, doi:10.1016/j.isci.2021.103112 (2021).

S10 Lin, Y. H. *et al.* Global Proteome and Phosphoproteome Characterization of Sepsis-induced Kidney Injury. *Mol Cell Proteomics* **19**, 2030-2047, doi:10.1074/mcp.RA120.002235 (2020).

S11 Leman, J. K. *et al.* Macromolecular modeling and design in Rosetta: recent methods and frameworks. *Nat Methods* **17**, 665-680, doi:10.1038/s41592-020-0848-2 (2020).

S12 Maier, J. A. *et al.* ff14SB: Improving the Accuracy of Protein Side Chain and Backbone Parameters from ff99SB. *J Chem Theory Comput* **11**, 3696-3713, doi:10.1021/acs.jctc.5b00255 (2015).

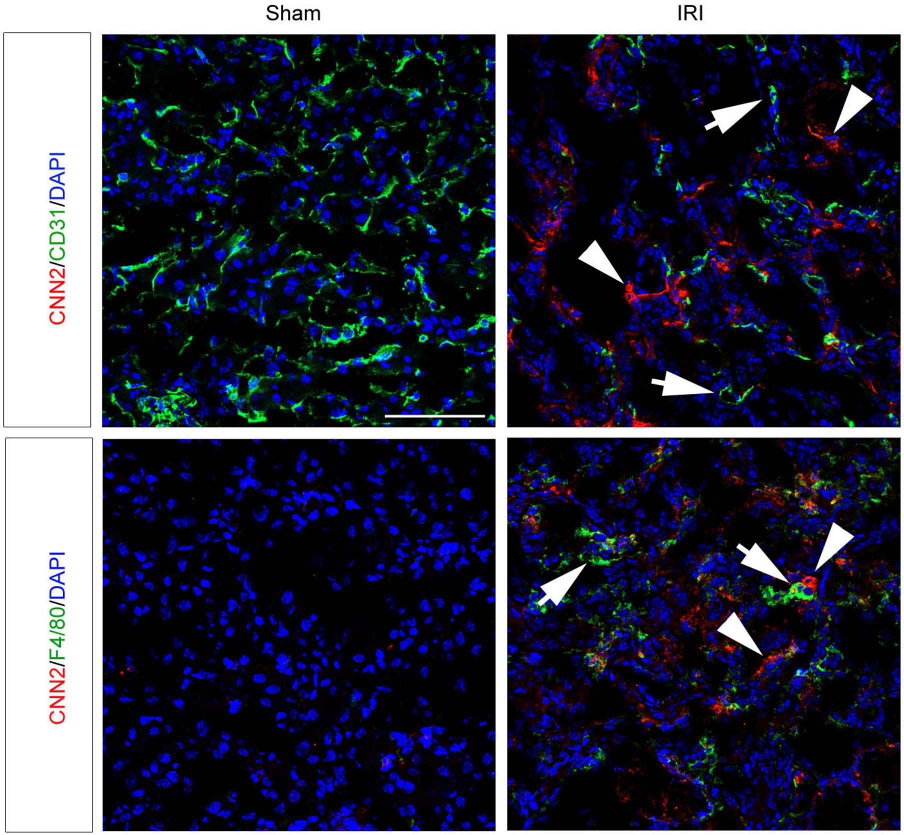

Supplementary Figure S1: Cell specificity of CNN2 expression in the ischemic kidneys. Representative confocal images showed the colocalization of CNN2 and CD31 (Upper panel) or F4/80 (Lower panel) in the ischemic kidneys. Arrows indicate CD31+ or F4/80+ cells. Arrowheads indicate CNN2+ cells. Scale bar, 50 µm.

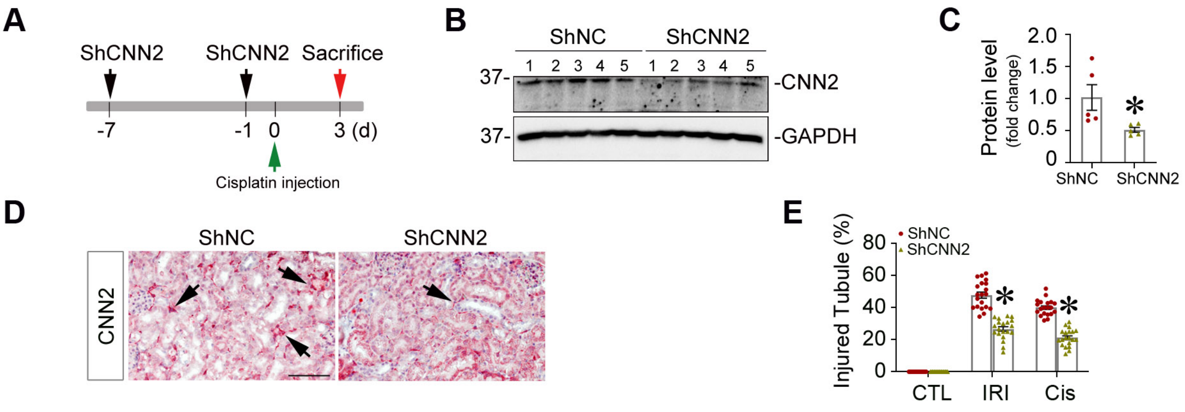

Supplementary Figure S2: Knockdown of CNN2 protects against AKI induced by Cisplatin. (**A**) Schematic diagram shows the strategies for ShCNN2 administration and a mouse AKI model construction induced by cisplatin. (**B, C**) Western blot assay demonstrated CNN2 protein expression in the kidneys of ShNC and ShCNN2 mice at 3 days after cisplatin injection (**B**) and quantitative data was presented (**C**). Numbers indicate individual animals within each group. Graphs are presented as means ± SEM. * *P* < 0.05 (n=5). (**D**) Immunohistochemical staining showed CNN2 expression and distribution in the kidneys of ShNC and ShCNN2 mice at 3 days after cisplatin injection. Scale bar, 50 μm. Arrows indicate positive staining. (**E**) The quantitative data of kidney morphological changes in ShNC and ShCNN2 mice after ischemic AKI at 1 day or cisplatin-induced AKI at 3 days. * *P* < 0.05 (n = 5, 4 random images were selected per mouse, each dot represents the score of the according image). Differences between groups were analyzed using unpaired t tests or ANOVA followed by the Student-Newman-Keuls test.

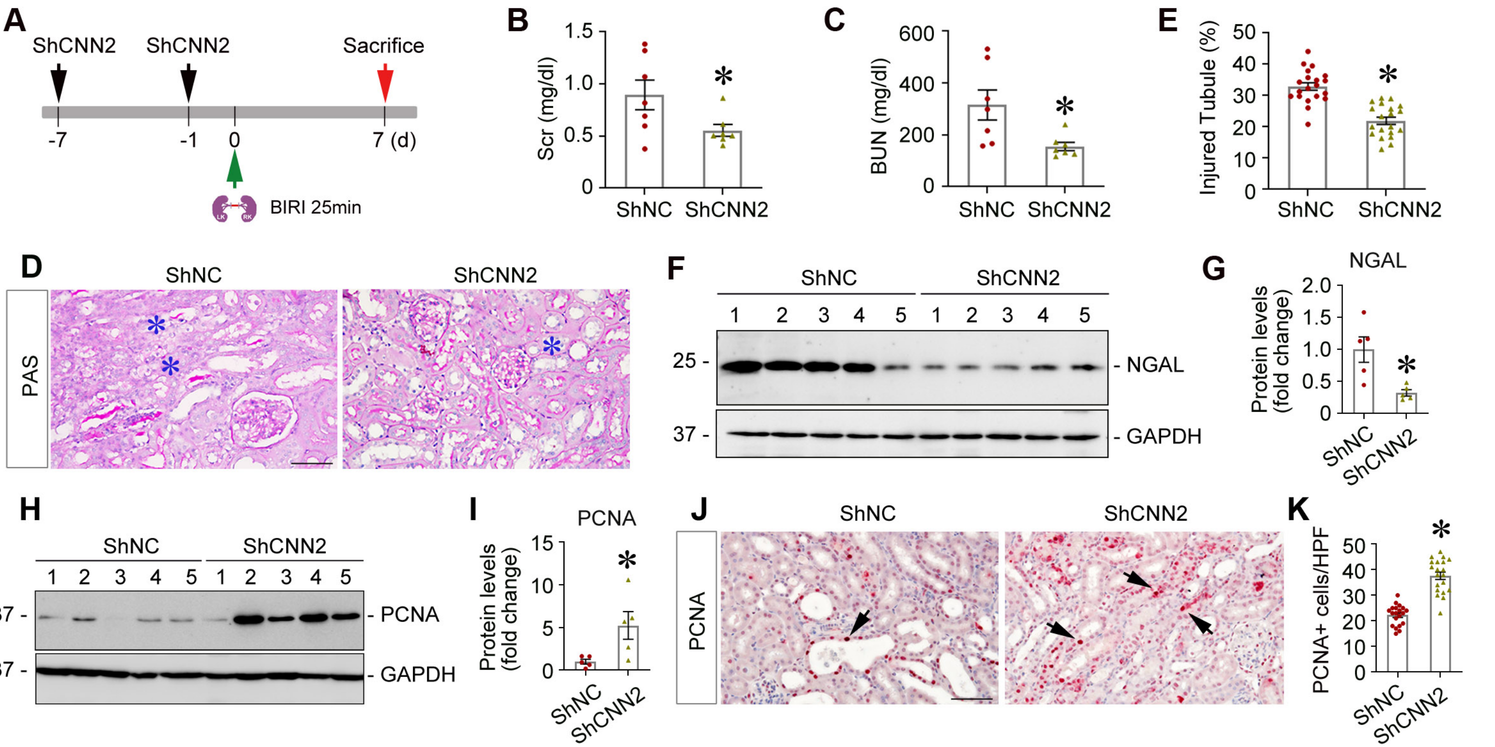

Supplementary Figure S3: Knockdown of CNN2 improves AKI repair after ischemic AKI. (**A**) Schematic diagram shows the strategies for ShCNN2 administration and a moderate (25 minutes) mouse AKI model construction induced by bilateral IRI. (**B, C**) Serum creatinine (Scr) and blood urea nitrogen (BUN) levels in ShNC and ShCNN2 mice at 7 days after IRI. * $P < 0.05$ (n=7). (**D, E**) Periodic acid–Schiff (PAS) staining showed kidney morphological changes of ShNC and ShCNN2 mice at 7 days after IRI. The quantitative data was presented (**E**). Scale bar, 50 μm. Blue asterisks indicate injured tubules. (**F, G**) Representative western blot (**F**) and quantitative data (**G**) of NGAL expression in ShNC and ShCNN2 mice kidneys at 7 days after IRI. Numbers indicate individual animals within each group. * $P < 0.05$ (n=5). (**H, I**) Representative western blot (**H**) and quantitative data (**I**) of PCNA expression in ShNC and ShCNN2 mice kidneys at 7 days after IRI. Numbers indicate individual animals within each group. * $P < 0.05$ (n=5). (**J**) Immunohistochemical staining showed PCNA expression and distribution in the kidneys of ShNC and ShCNN2 mice at 7 days after IRI. Scale bar, 50 μm. Arrows indicate positive staining. (**E**) The quantitative data of PCNA staining. * $P < 0.05$ (n = 5, 4 random images were selected per mouse, each dot represents the score of the according image). Graphs are presented as means ± SEM. Differences between groups were analyzed using unpaired t tests.

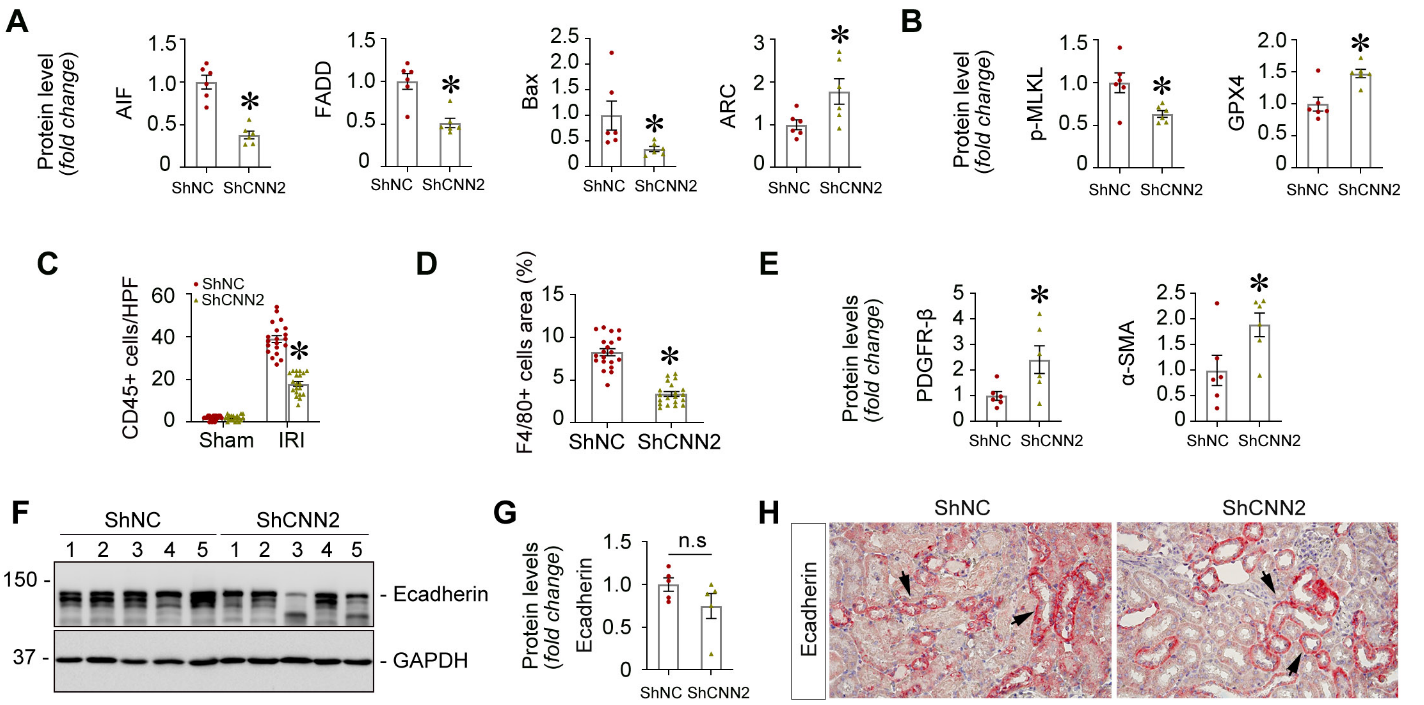

Supplementary Figure S4: Knockdown of CNN2 reduced cell apoptosis and inflammation after IRI. (**A**, **B**) Quantitative data for AIF, FADD, Bax, ARC, p-MLKL, and GPX4 after IRI at 1 day. Graphs are presented as means ± SEM. * $P < 0.05$ (n=6). (**C, D**) Quantitative data for immunostaining of CD45 (**C**) and F4/80 (**D**) in the kidneys at 1 day after IRI. * $P < 0.05$ (n = 5, 4 random images were selected per mouse, each dot represents the score of the according image). (**E**) Quantitative data for PDGFR-β and α-SMA after IRI at 1 day. * $P < 0.05$ (n=6). (**F, G**) Representative western blot (**F**) and quantitative data (**G**) for E-cadherin expression in ShNC and ShCNN2 kidney at 1 day after IRI. (n=5). (**H**) Immunohistochemical staining for E-cadherin in ShNC and ShCNN2 kidney at 1 day after IRI. Arrows indicate positive staining. IRI, ischemia reperfusion injury; AIF, apoptosis inducing factor; ARC, apoptosis repressor with caspase recruitment domain; FADD, fas-associated protein with death domain; p-MLKL, phosphor-mixed lineage kinase domain-like protein; GPX4, glutathione peroxidase 4. PDGFR-β, platelet derived growth factor receptor β; α-SMA, α-smooth muscle actin. Graphs are presented as means ± SEM. n.s., not statistically significant. Differences between groups were analyzed using unpaired t tests or ANOVA followed by the Student-Newman-Keuls test..

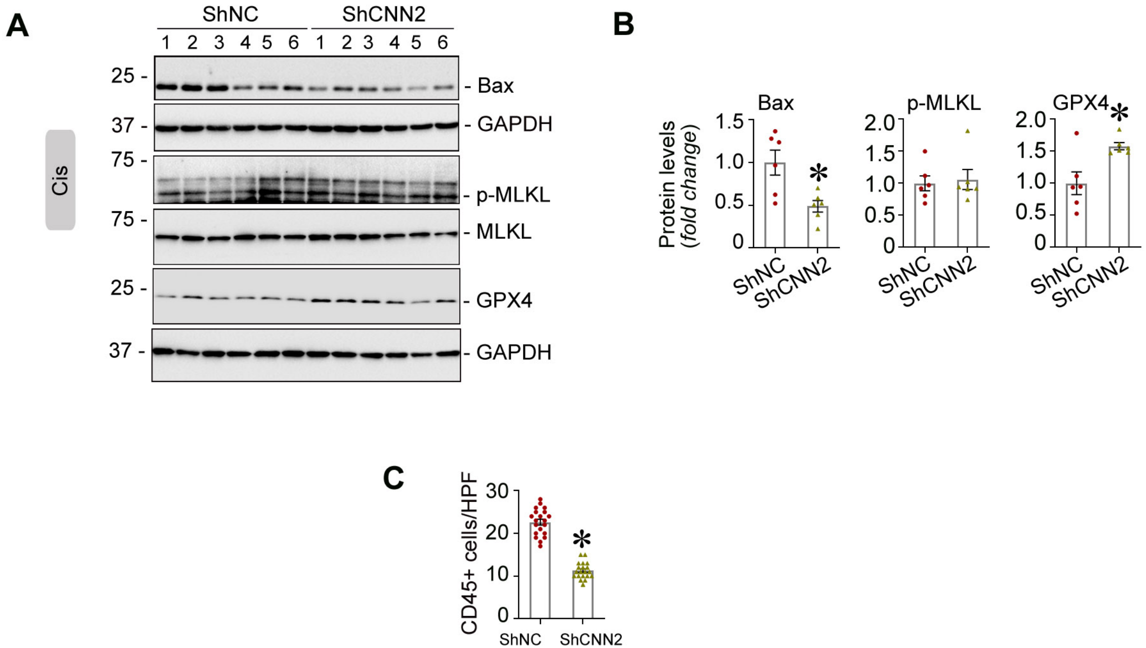

Supplementary Figure S5: Knockdown of CNN2 alleviated cell death and inflammation in AKI induced by Cisplatin. (**A, B**) Western blot assay demonstrated the expression of Bax, p-MLKL, and GPX4 in the kidneys of ShNC and ShCNN2 mice at 3 days after cisplatin injection (**A**) and quantitative data was presented (**B**). Numbers indicate individual animals within each group. * $P < 0.05$ (n=6). (**C**) Quantitative data for CD45 expression in the kidneys from ShNC and ShCNN2 mice at 3 days after cisplatin injection. * $P < 0.05$ (n=5). p-MLKL, phosphor-mixed lineage kinase domain-like protein; MLKL, mixed lineage kinase domain-like protein; GPX4, glutathione peroxidase 4. Graphs are presented as means ± SEM. Differences between groups were analyzed using unpaired t tests.

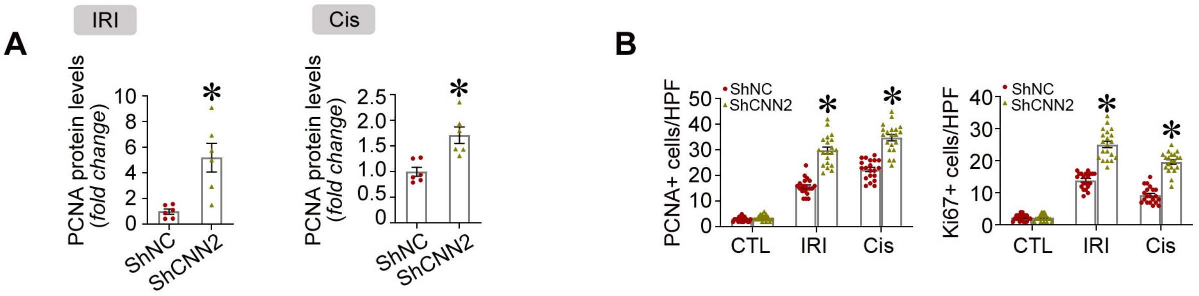

Supplementary Figure S6: Knockdown of CNN2 promotes tubular cell proliferation after AKI. (**A**) Quantitative data of PCNA protein in the kidneys of ShNC and ShCNN2 mice at 1 day after IRI or 3 days after cisplatin injection. * $P < 0.05$ (n=6). (**B**) Quantitative data of PCNA- and Ki67-positive tubular cells in the kidneys of ShNC and ShCNN2 mice at 1 day after IRI or 3 days after cisplatin injection. * $P < 0.05$ (n=5, 4 random images were selected per mouse, each dot represents the score of the according image). PCNA, proliferating cell nuclear antigen. Graphs are presented as means ± SEM. Differences between groups were analyzed using unpaired t tests or ANOVA followed by the Student-Newman-Keuls test.

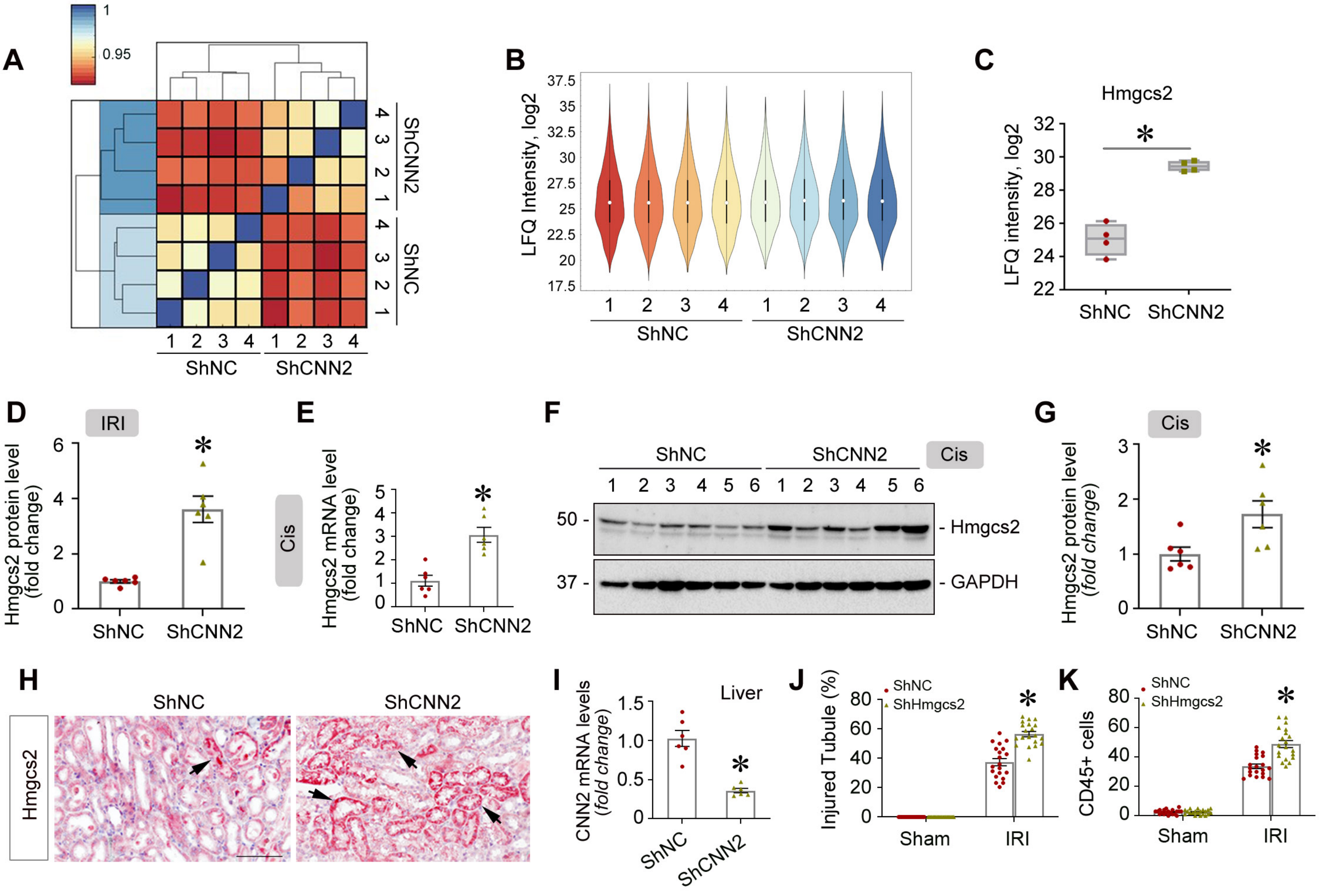

Supplementary Figure S7: Knockdown of CNN2 induces Hmgcs2 after AKI. (**A**) Correlation of kidney proteome profiles between ShNC and ShCNN2 mice after ischemic AKI. Color scale represents R2 values. (**B**) Violin plot of ANOVA significant proteins (Permutation FDR 0.05) among ShNC and ShCNN2 mice injured kidneys after ischemic AKI. Label-free quantitation (LFQ) intensities of represented proteins were z-scored and plotted according to the color bar. (**C**) The intensity plot shows Hmgcs2 level among ShNC and ShCNN2 mice at 1 day after IRI. Box plots with median, min, max, and interquartile range. * P < 0.05 (n=4). (**D**) Quantitative data of kidney Hmgcs2 protein after IRI. * *P* < 0.05 (n=6). (**E**) Quantitative real-time PCR (qPCR) showed the level of Hmgcs2 mRNA in the kidneys of ShNC and ShCNN2 mice at 3 days after cisplatin injection. * *P* < 0.05 (n=6). (**F**, **G**) Western blots assay demonstrated the level of Hmgcs2 protein in the kidneys of ShNC and ShCNN2 mice at 3 days after cisplatin injection (**F**) and quantitative data was presented (**G**). Numbers indicate individual animals within each group. Graphs are presented as means ± SEM. * *P* < 0.05 (n=6). (**H**) Immunohistochemical staining showed Hmgcs2 expression in the kidneys of ShNC and ShCNN2 mice at 3 days after cisplatin injection. Scale bar, 50 μm. Arrows indicate positive staining. (**I**) qPCR analysis showed that CNN2 mRNA levels were reduced in the liver after knockdown of CNN2 employing hydrodynamic gene delivery technique. * *P* < 0.05 (n=6). (**J, K**) The quantitative data for Periodic Acid Schiff (**J**) and CD45 (**K**) staining of ShNC and ShHmgcs2 mice kidneys at 1 day after IRI. * *P* < 0.05 (n = 5, 4 random images were selected per mouse, each dot represents the score of the according image). Hmgcs2, 3-hydroxy-3-methylglutaryl-CoA synthase 2. IRI, ischemia reperfusion injury. Graphs are presented as means ± SEM. Differences between groups were analyzed using unpaired t tests or ANOVA followed by the Student-Newman-Keuls test.

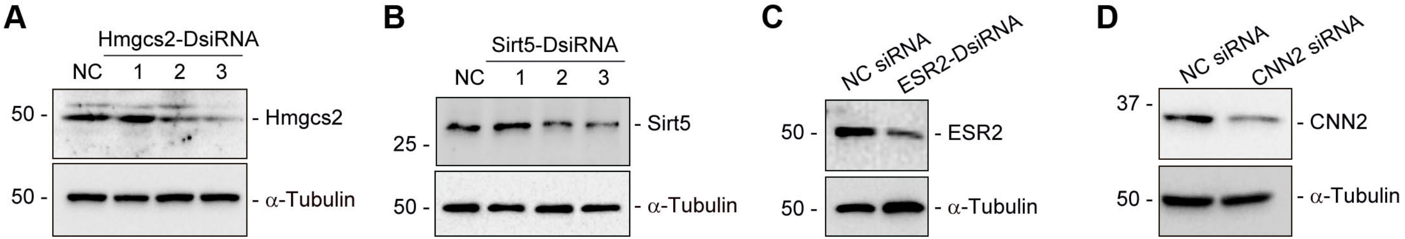

Supplementary Figure S8: CNN2 regulates Hmgcs2 expression in vitro. (**A**-**C**) Western blot analyses demonstrated the efficiency of knockdown Hmgcs2 (**A**), sirt5 (**B**) or ESR2 (**C**) in normal rat kidney epithelial cells (NRK-52E) after transfected with dicer-substrate siRNA. (**D**) Western blot assay demonstrated the efficiency of knockdown CNN2 in normal rat kidney fibroblasts (NRK-49F) after transfected with siRNA. Sirt5, sirtuin-5; ESR2, estrogen receptor 2; Hmgcs2, 3-hydroxy-3-methylglutaryl-CoA synthase 2.

A

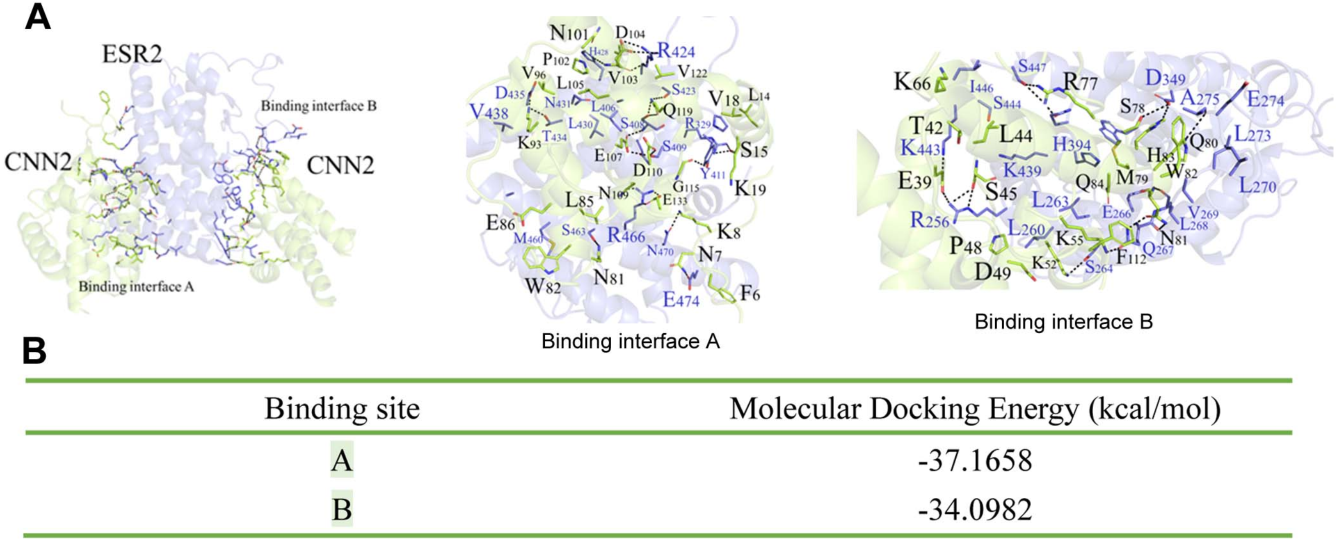

B

Binding site	Molecular Docking Energy (kcal/mol)
A	-37.1658
B	-34.0982

Supplementary Figure S9: Molecular docking analysis shows the binding patterns between CNN2 and ESR2. (**A**) The selected two binding sites between CNN2 and ESR2 proteins form a dense hydrogen bonding network system, which play critical roles in the stable binding between two proteins. The binding energy of the two binding sites are presented (**B**).

Supplementary Table S1. Nucleotide sequences of the primers used for qRT-PCR

gene	Primer Sequence 5' to 3'	
	Forward	Reverse
CNN2 (M)	AGGAAGCAGAACTCCGAAGC	CCAGTTCTGCATAGAGCGGT
Hmgcs2 (M)	AGCTACTGGGATGGTCGCTA	ACGCGTTCTCCATGTGAGTT
PPARα (M)	ACCACTACGGAGTTCACGCATG	GAATCTTGCAGCTCCGATCACAC
FOXA2(M)	CGAGCACCATTACGCCTTCAAC	AGTGCATGACCTGTTCGTAGGC
FGF21 (M)	ATCAGGGAGGATGGAACAGTGG	AGCTCCATCTGGCTGTTGGCAA
Sirt2 (M)	CGAAGGAGTGACACGCTACATG	GGTGGTACTTCTCCAGGTTTGC
Sirt3 (M)	GCTACATGCACGGTCTGTCGAA	CAATGTCGGGTTTCACAACGCC
Sirt5 (M)	ATCGCAAGGCTGGCACCAAGAA	CTAAAGCTGGGCAGATCGGACT
Rantes (M)	GCTGCTTTGCCTACCTCTCC	TCGAGTGACAAACACGACTGC
IL-6 (M)	CTTGGGACTGATGCTGGTG	TCCACGATTTCCCAGAGAAC
TNFα (M)	CCCTCACACTCAGATCATCTTCT	CCCTCACACTCAGATCATCTTCT
MCP1 (M)	TTAAAAACCTGGATCGGAACCAA	GCATTAGCTTCAGATTTACGGGT
β-actin (M)	CAGCTGAGAGGGAAATCGTG	CGTTGCCAATAGTGATGACC

Supplementary Table S2. The information of the applied primary and secondary antibodies

Name	Vendor	Category Number	Application
CNN2	Proteintech Group, Rosemont, IL	21073-1-AP	WB, IF, IHC
PPARα	Proteintech Group, Rosemont, IL	15540-1-AP	WB
Sirt5	Proteintech Group, Rosemont, IL	15122-1-AP	WB, IHC
ESR2	Proteintech Group, Rosemont, IL	14007-1-AP	WB
PDGFR-β	Invitrogen, Waltham, MA	14-1402-82	IF
F4/80	Invitrogen, Waltham, MA	14-4801-82	IF
Hmgcs2	LsBio, Seattle, WA	LS-B11023	WB, IF, IHC
NGAL	Abcam, Cambridge, MA	ab63929	WB
P-MLKL	Abcam, Cambridge, MA	ab196436	WB, IHC
GPX4	Abcam, Cambridge, MA	ab125066	WB, IHC
PCNA	Cell signaling Technology, Danvers, MA	#13110	WB, IHC
Ki67	Cell signaling Technology, Danvers, MA	#12202	IHC
Cleaved Caspase-3	Cell signaling Technology, Danvers, MA	#9664	WB, IF
AIF	Cell signaling Technology, Danvers, MA	#5318	WB
ARC	Cell signaling Technology, Danvers, MA	#38916	WB
MLKL	Cell signaling Technology, Danvers, MA	#37705	WB
Caspase3	Cell signaling Technology, Danvers, MA	#9662	WB
Cyclin D1	Cell signaling Technology, Danvers, MA	#55506	WB
Ecadherin	Cell signaling Technology, Danvers, MA	#3195	WB, IHC
CD45	Cell signaling Technology, Danvers, MA	#70257	IHC
PDGFR-β	Cell signaling Technology, Danvers, MA	#3169	WB
Succinyllysine	PTM Biolabs, Chicago, IL	PTM-401	WB
FADD	Sigma, St. Louis, MO	05-486	WB
α-Tubulin	Sigma, St. Louis, MO	T9026	WB
Hmgcs2	Santa Cruz Biotechnology, Dallas, Texas	sc-393256	Co-IP
Bax	Santa Cruz Biotechnology, Dallas, Texas	sc-7480	WB
GAPDH	Santa Cruz Biotechnology, Dallas, Texas	sc-32233	WB
CD31	BD Biosciences, Franklin Lakes, NJ	550274	IF
Anti-Mouse IgG	Abcam, Cambridge, MA	ab6789	WB
Cy3 Anti-Rabbit	Jackson ImmunoResearch	711-165-152	IF
Alexa Fluor® 488 Anti-Rabbit	Jackson ImmunoResearch	711-545-152	IF
Biotin-Anti-Mouse	Jackson ImmunoResearch	715-065-150	IHC
Biotin-Anti-Rabbit	Jackson ImmunoResearch	711-065-152	IHC

WB, western blot; IHC, Immunohistochemical staining; IF, Immunofluorescence staining; Co-IP, Co-immunoprecipitation
